# Bactofilins are essential spatial organizers of peptidoglycan insertion in the Lyme disease spirochete *Borrelia burgdorferi*

**DOI:** 10.1128/jb.00198-26

**Published:** 2026-06-29

**Authors:** Christopher B. Zinck, Valentina Carracoi, Zachary A. Kloos, Jenny Wachter, Cindi L. Schwartz, Philip E. Stewart, Christine Jacobs-Wagner, Patricia A. Rosa, Constantin N. Takacs

**Affiliations:** 1 Department of Biology, College of Science, Northeastern University 1848, Boston, Massachusetts, USA; 2 Laboratory of Bacteriology, Rocky Mountain Laboratories, Division of Intramural Research, National Institute of Allergy and Infectious Diseases, National Institutes of Health, Hamilton, Montana, USA; 3 Microbiology Program, Yale University 5755, New Haven, Connecticut, USA; 4 Electron Microscopy Unit, Research Technologies Branch, Division of Intramural Research, National Institute of Allergy and Infectious Diseases, National Institutes of Health, Hamilton, Montana, USA; 5 Laboratory of Virology, Rocky Mountain Laboratories, Division of Intramural Research, National Institute of Allergy and Infectious Diseases, National Institutes of Health, Hamilton, Montana, USA; 6 Sarafan ChEM-H Institute, Stanford University 6429, Stanford, California, USA; 7 Department of Biology, Stanford University 6429, Stanford, California, USA; 8 Howard Hughes Medical Institute, Stanford University 6429, Stanford, California, USA; 9 Department of Microbiology and Immunology, Stanford University School of Medicine 10624 , Stanford, California, USA; University of Florida Department of Microbiology and Cell Science, Gainesville, Florida, USA

**Keywords:** bactofilin, peptidoglycan, *Borrelia*, Lyme disease, spirochetes

## Abstract

The Lyme disease spirochete *Borrelia burgdorferi* has a distinctive pattern of growth. Newly born cells elongate by primarily inserting peptidoglycan at mid-cell, while in longer cells, additional insertion sites form at the one-quarter and three-quarter positions along the cell length. It is not known how peptidoglycan insertion is concentrated at these locations in *B. burgdorferi*. In other bacteria, multi-protein complexes are known to synthesize new peptidoglycan, and are often organized by cytoskeletal proteins. We show here that *B. burgdorferi*’s zonal concentration of peptidoglycan insertion requires BB0538 (BbbF) and BB0245 (BbbG), two members of the bactofilin class of cytoskeletal proteins. Bactofilin depletion redistributed peptidoglycan insertion along the cell length. Prolonged bactofilin depletion arrested growth in culture and induced extensive cell blebbing, indicating that *B. burgdorferi* bactofilins are essential for viability. Fluorescent protein fusions of BbbF and BbbG localized to new zones of growth before peptidoglycan insertion occurred at these sites, with BbbG localization dependent on BbbF. Our results show that BbbF and BbbG direct the spatial patterning of new peptidoglycan insertion in *B. burgdorferi*.

**IMPORTANCE:**

The spirochetal bacterium *Borrelia burgdorferi* causes Lyme disease, the most prevalent vector-borne infection in North America and Europe. Cellular replication, which requires growth and division of the peptidoglycan cell wall, facilitates *B. burgdorferi* transmission to, and dissemination within, new hosts. Cellular replication is therefore essential for pathogenesis. Bactofilins regulate peptidoglycan-related processes in several bacteria but are typically non-essential for cellular replication. Bactofilin-encoding genes can be readily deleted in multiple bacterial species. In contrast, we show that the *B. burgdorferi* bactofilins BbbF and BbbG are essential for cellular viability and direct zonal peptidoglycan insertion. Our findings broaden the spectrum of known bactofilin functions and advance our understanding of how peptidoglycan insertion is regulated in this unusual, medically important spirochetal bacterium.

## INTRODUCTION

*Borrelia burgdorferi* and related spirochetal bacteria are zoonotic pathogens vectored by ixodid ticks (1, 2). These bacteria cause Lyme disease, also known as Lyme borreliosis, the most prevalent vector-borne infectious disease in temperate climates of the Northern Hemisphere (3–6). Larval ticks typically acquire *B. burgdorferi* when they feed on an infected vertebrate animal and then maintain the spirochetes through both the larva-to-nymph and nymph-to-adult molts (7, 8). Ticks transmit *B. burgdorferi* to vertebrate hosts during post-acquisition feedings (9, 10). In the host, *B. burgdorferi* disseminates from the skin to multiple tissues, including distant skin sites, the heart, joints, and the meningeal lining of the central nervous system (11–16). Lyme borreliosis can present with generalized or localized symptoms that include fever, malaise, skin rash, heart block and arrhythmias, meningitis, Bell’s palsy, and arthritis (3, 17–21). While antibiotic treatment is a generally effective therapy (22–24), post-infectious symptoms can persist in 10–20% of patients and can be debilitating (25–27).

*B. burgdorferi* cellular replication is required for the spirochete’s colonization of and persistence in its tick vectors and vertebrate hosts. The bacterium replicates in the tick midgut during tick feeding and digestion of the blood meal (7, 8, 28), and likely even in post-molt unfed nymphs (29, 30). In the host, *B. burgdorferi* replication underpins its effective dissemination from the bite site, colonization of distal tissues, and long-term antigenic variation-mediated evasion of the host adaptive immune response (14, 31–36). Bacterial replication requires regulated growth and division of the peptidoglycan sacculus, a mesh-like macromolecular polymer made of glycan chains cross-linked by short stem peptides (37–39). Peptidoglycan lies outside the cytoplasmic membrane, opposes the turgor pressure of the cytoplasm, and helps maintain cellular shape and integrity (38). In spirochetes, both peptidoglycan and motility-generating flagella are found within the periplasmic space delimited by the inner and outer membranes (40–50).

Previous studies in several model bacteria have identified two widely conserved protein complexes that carry out peptidoglycan biosynthesis and attendant cell growth. The ubiquitous bacterial divisome complex synthesizes a cell wall septum that separates the two offspring cells during cytokinesis (37, 51–54). Additionally, in many rod-shaped bacteria, the elongasome contributes to cell growth by inserting new peptidoglycan along the sidewall (37, 38, 55–58). Alternatively, growth can occur through elongasome-independent polar synthesis of new peptidoglycan (59–65). Both the divisome and the elongasome are organized by bacterial cytoskeletal elements (66) and contain transglycosylases (e.g., FtsW or RodA, respectively), transpeptidases (e.g., FtsI/PBP3 or PBP2), and regulatory, non-enzymatic components (e.g., FtsN or MreCD) (37, 67, 68). The tubulin homolog FtsZ governs assembly of the divisome (52, 54, 69), while the actin homolog MreB orients the activity of the elongasome perpendicular to the long axis of rod-shaped bacteria (56–58). Additionally, bacterial intermediate filament proteins can modulate cell growth (70), for example, by associating with the plasma membrane and exerting a mechanical force that directs preferential peptidoglycan insertion on the opposite sidewall (66, 71, 72).

Another class of cytoskeletal proteins broadly represented among bacteria, archaea, and some eukaryotes is the bactofilins (73–75). They share a triangular, parallel beta-barrel structure formed by the conserved domain of unknown function (DUF) 583 (73, 75, 76). This structure can assemble head-to-head and tail-to-tail in the absence of any cofactor to yield nonpolar protofilaments approximately 3 nm wide (73). These protofilaments can, in turn, interact laterally to form bundles of varying thickness or two-dimensional crystalline lattices (73, 77, 78). The DUF583 bactofilin domain is flanked by N- and C-terminal tails of varying length that mediate protein-protein interactions and membrane association (73, 77, 79, 80).

Bactofilins serve as scaffolds for diverse cellular processes, including motility, biofilm formation, establishment and maintenance of cell polarity, and chromosome segregation (75, 81–84). They have also been suggested or demonstrated to participate in cell growth and morphogenesis processes, such as cell division (75) and cell size regulation (85), respectively. In other bacteria, bactofilins control the growth and shape of cell appendages, such as stalks or hyphae (75, 79, 86), or a peculiar mode of cell growth whereby new cells bud from the free end of a stalk (87). Disruption of bactofilin function has also been shown to induce kinks in otherwise straight rod-shaped cells (88, 89), modify the cell curvature radius (87–90), or change a cell’s helical pitch (91). These findings established bactofilins as key modulators of the bacterial rod shape.

The mechanisms underpinning bactofilin function remain poorly understood, but several patterns have begun to emerge. Membrane association and filament formation are essential to the function of some bactofilins (73, 80, 92). Additionally, evidence exists of a phylogenetically conserved, functional interaction between some bactofilins and M23 endopeptidases, which are peptidoglycan hydrolases (79, 87, 90, 93). In *Hyphomonas neptunium* and *Rhodospirillum rubrum*, bactofilin BacA recruits an M23 endopeptidase, LmdC, to modify cell shape (87). In *Helicobacter pylori*, the bactofilin CcmA modifies the extent of peptidoglycan crosslinking, possibly through indirect regulation of the stability of the M23 peptidase Csd1 (90, 94, 95). In contrast, bactofilin BacA of *Caulobacter crescentus* interacts with a peptidoglycan synthase, PbpC (75). This finding raises the possibility that bactofilins may regulate peptidoglycan synthases in the other species in which they are known to influence peptidoglycan insertion (89, 96).

*B. burgdorferi* cells have a distinctive pattern of growth. While peptidoglycan insertion occurs at low levels along most of the cell body, except at the inert poles, notably higher insertion occurs at mid-cell (97, 98). As the cells grow, new zones of localized insertion develop at the one-quarter and three-quarter cell positions, and the mid-cell zone becomes a division site (97). This pattern of zonal cell wall insertion establishes, in one generation, the primary growth locations and eventual division sites of the next generation of cells (97). A CRISPR interference-based gene expression knockdown approach implicated the elongasome in peptidoglycan synthesis at these locations, as knockdown of *mreB* and *rodA* expression caused localized bulging (a typical elongasome inhibition phenotype) at the one-quarter, mid-cell, and three-quarter positions (99). Additionally, the early divisome protein FtsA localizes to these sites, where it precedes peptidoglycan insertion (100). Given the paucity of knowledge concerning mechanisms of cell growth in *B. burgdorferi* and the finding that spirochete *Leptospira biflexa* employs bactofilin LbbD in regulating cell helical pitch, cell wall strength, and motility (91), we investigated the functions of two *B. burgdorferi* bactofilins using genetic and imaging approaches.

## RESULTS

### *B. burgdorferi* encodes three bactofilins

Several studies have reported that spirochetes encode bactofilins (73, 75, 87, 91). The *Leptospiraceae* encode five paralogs, LbbA through LbbE, of which LbbD has been characterized (91). Using Basic Local Alignment Search Tool (BLAST) searches, we identified 879 unique spirochete bactofilin sequences, which we assembled into a phylogenetic tree that contained the five *Leptospiraceae* clusters, as well as three *Borreliaceae*-encoded bactofilin clusters (Fig. S1). One cluster, which contains protein BB0231 encoded by *B. burgdorferi* strain B31, appeared related to the *Leptospiraceae* bactofilin cluster containing LbbA (Fig. S1). Therefore, we propose renaming BB0231 as *B. burgdorferi* bactofilin A or BbbA. The other two clusters contained proteins BB0538 and BB0245, respectively, and did not closely relate to the remaining *Leptospiraceae* bactofilin clusters (Fig. S1). We therefore propose renaming these bactofilins as BbbF and BbbG, respectively. Within the *Borreliaceae*, the bactofilin loci were well conserved among the 28 Lyme disease spirochete genomes visualized using the BorreliaBase online resource (101) (Fig. S2A). Gene *bbbA* is flanked by *hbb* (*bb0232*), encoding a DNA-binding protein (102, 103), and the *bb0230* gene encoding the transcription termination factor Rho (Fig. S2A). Both *bbbF* and *bbbG* are flanked by uncharacterized genes, for which the predicted functions annotated in the B31 genome are stated in Fig. S2A. Analysis of total RNA revealed that all three bactofilins were expressed in culture (Fig. S2B and C).

The *Borreliaceae* bactofilin sequences contain a conserved central bactofilin domain (Fig. S3A, gray bar, and Fig. S3B) that aligned well with the central domains of previously studied bactofilins from divergent species (Fig. S4A). Moreover, the bactofilin domains of BbbF and BbbG contained well-conserved glycine residues (Fig. S3B and S4A), which are defining sequence features of the bactofilin fold (104). Indeed, AlphaFold 3 (105) structural modeling of BbbA, BbbF, and BbbG predicted that the central domain of each protein folds into a bactofilin-characteristic triangular beta-barrel (Fig. S4B through D) (76, 78, 104). This central bactofilin domain was flanked by N- and C-terminal tails predicted to be less structured (Fig. S4B through D). While a subset of the BbbF proteins had a predicted N-terminal tail extension of 14 amino acids (29 out of 79) or 16 amino acids (1 out of 79) (Fig. S3B, star), alignment of all BbbF sequences revealed that these longer sequences also have a conserved internal methionine residue (Fig. S3C, purple arrowhead) that aligns with the initiating methionine of the shorter sequences. Moreover, a closer look at the genome of the *B. burgdorferi* type strain B31 revealed that the codon encoding the annotated M15 residue of BbbF is preceded by a likely ribosome binding site (RBS, Fig. S3D, blue letters) with a nucleotide sequence (5′-AGAGG-3′) similar to that of the consensus *B. burgdorferi* RBS*,* 5′-AGGAG-3′ (106). In contrast, we could not discern a likely RBS upstream of the annotated START codon of the same *bbbF* gene, leading us to propose that the BbbF sequence begins at the annotated M15 residue. Hereafter, we report the results of our investigations of BbbF and BbbG and reserve the investigation of BbbA, which appears functionally divergent, for another study.

### BbbG is required for *B. burgdorferi* growth in culture and maintenance of cell morphology

To investigate bactofilin BbbG, we created the conditional mutant strain VCbb08 by placing the native *bbbG* gene under the control of the isopropyl β-D-1-thiogalactopyranoside (IPTG)-inducible promoter P*_flac_* (107) (Fig. S5). This strain also constitutively produces LacI (Fig. S5), which binds the *lacO* sequence within the P*_flac_* promoter and downregulates (but not necessarily abolishes) gene expression (107, 108). Addition of IPTG to the growth medium relieves LacI-mediated transcriptional repression (107, 108).

**Fig 1:**
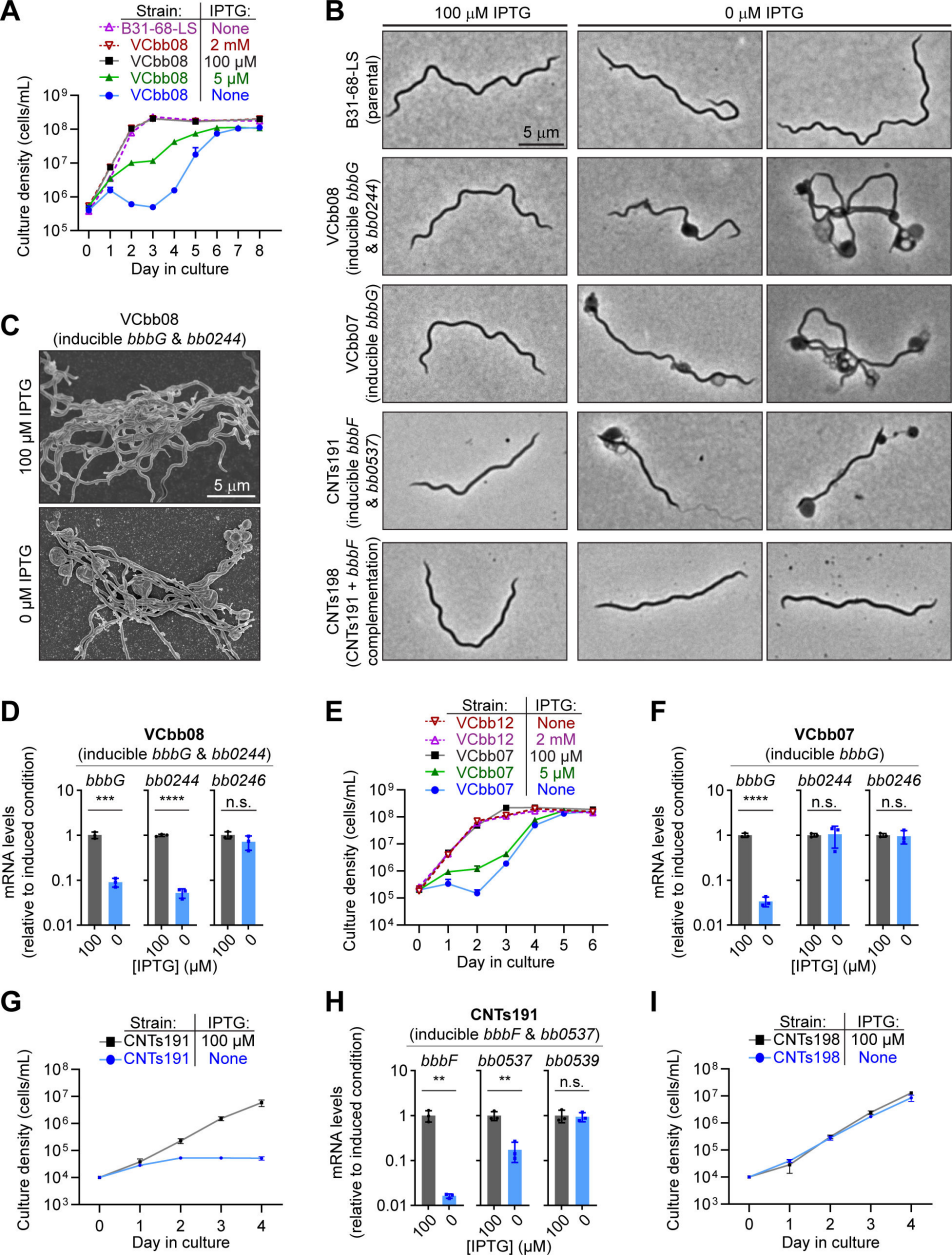
Characterization of growth, morphology, and gene expression effects of *bbbG* and *bbbF* expression knockdown. (**A**) Growth curves of parental strain B31-68-LS (violet) and derived strain VCbb08 (containing IPTG-inducible *bbbG* and *bb0244*) grown with the IPTG amounts indicated at the right. Shown are means ± standard deviations of results from *n* = 3 cultures. (**B**) Phase-contrast light micrographs of cells of control and bactofilin expression knockdown strains grown with 100 μM IPTG (left) or for 48 h without IPTG (0 μM IPTG, right). Strain identities are provided at the left. One image is provided for growth with IPTG, and two for growth without. (**C**) Scanning electron micrographs of cells of strain VCbb08 grown with 100 μM IPTG or for 48 h without IPTG. (**D**) Gene expression (mRNA) levels for *bbbG*, *bb0244*, and *bb0246* measured in strain VCbb08 grown with 100 μM IPTG or in the absence of IPTG for 24 h. Expression (individual values as well as means ± standard deviations from *n* = 3 cultures) of each gene is shown relative to the mean value for the same gene in the culture grown in the presence of IPTG. (**E**) Growth curves of strains VCbb12 (expressing its native *bbbG* gene and carrying a second, shuttle vector-borne, IPTG-inducible, *bbbG* copy) and VCbb07 (carrying only a shuttle vector-borne, IPTG-inducible *bbbG* copy) grown with the IPTG amounts indicated at the right. Shown are means ± standard deviations of results from *n* = 3 cultures. (**F**) Gene expression (mRNA) levels for *bbbG*, *bb0244*, and *bb0246* measured in strain VCbb07 grown with 100 μM IPTG or in the absence of IPTG for 24 h. Expression (individual values as well as means ± standard deviations from *n* = 3 cultures) of each gene is shown relative to the mean value for the same gene in the culture grown in the presence of IPTG. (**G**) Growth curves of strain CNTs191 (containing IPTG-inducible *bbbF* and *bb0537*) grown with 100 μM IPTG or without IPTG. Shown are means ± standard deviations of results from *n* = 3 cultures. (**H**) Gene expression (mRNA) levels for *bbbF*, *bb0537*, and *bb0539* measured in strain CNTs191 grown with 100 μM IPTG or in the absence of IPTG for 24 h. Expression (individual values as well as means ± standard deviations from *n* = 3 cultures) of each gene is shown relative to the mean value for the same gene in the culture grown in the presence of IPTG. (**I**) Growth curves of strain CNTs198 (conditional *bbbF* and *bb0537* expression strain complemented with shuttle vector-borne, constitutively expressed *bbbF*) grown with 100 μM IPTG or without IPTG. Shown are means ± standard deviations of results from *n* = 3 cultures. (**D**, **F**, and **H**) *P*-values (two-tailed, unpaired *t* test): ****, *P* < 0.0001; ***, *P* < 0.001; **, *P* < 0.01; n.s., not significant, *P* > 0.05.

In the presence of 2 mM or 100 μM IPTG, cultures of strain VCbb08 yielded growth curves nearly indistinguishable from those of the uninduced parental strain B31-68-LS (Fig. 1A), whose growth was unaffected by 2 mM IPTG (Fig. S6A). Cells of VCbb08 also had typical spirochetal cell morphology when grown with 100 µM IPTG (Fig. 1B and C). In contrast, incubation of VCbb08 in culture medium supplemented with 5 µM IPTG or lacking IPTG impaired spirochete growth (Fig. 1A). Incubation in IPTG-free media also caused severe membrane blebbing at varied locations along the cell length within 2 days (Fig. 1B and C). These cultures eventually resumed growth (Fig. 1A), which we traced, using whole-genome sequencing, to the presence of a LacI-inactivating mutation (Fig. S6B). Mutations inactivating LacI-mediated gene expression repression were previously reported when this system was used for conditional expression of essential genes in *B. burgdorferi* (109, 110).

Incubating VCbb08 without IPTG for 24 h decreased transcript levels of *bbbG* and the downstream gene *bb0244* by more than 90%, while expression of the upstream gene *bb0246* (Fig. S2A) was unaffected (Fig. 1D). This indicates that *bbbG* and *bb0244* are part of an operon and that downregulating the expression of either gene could be responsible for the growth and morphology defects we observed (Fig. 1A through C). To help distinguish between the contributions of *bbbG* and *bb0244* to these phenotypes, we first introduced a shuttle vector carrying an IPTG-inducible copy of *bbbG* into strain B31-68-LS (Fig. S5). The resulting strain, VCbb12, displayed similar growth kinetics in the presence or absence of 100 µM IPTG (Fig. 1E). In this background, we deleted the chromosomal *bbbG* copy, yielding strain VCbb07 that carries its sole *bbbG* copy on the resident shuttle vector under IPTG-inducible control (Fig. S5). Strain VCbb07 displayed wild-type growth in the presence of 100 μM IPTG, while its growth was impaired at 5 or 0 μM IPTG (Fig. 1E). As with strain VCbb08, growth of strain VCbb07 resumed after prolonged incubation without IPTG (Fig. 1E), which we traced to a mutation in the P*_flac_* promoter that removed the *lacO* sequence, likely preventing LacI-mediated repression (Fig. S6C). Moreover, culturing strain VCbb07 in the absence of IPTG lowered *bbbG* transcript levels within 24 h, but, in contrast with strain VCbb08, did not affect *bb0246* expression (Fig. 1D and F). Within 48 h of incubation without IPTG, strain VCbb07 also displayed severe blebbing (Fig. 1B), indicating that knockdown of *bbbG* expression alone caused the growth and morphology defects we documented. Taken together, our results indicate that *bbbG* is essential for *B. burgdorferi* growth in culture and maintenance of the cells’ typical morphology.

### BbbF is also required for *B. burgdorferi* growth in culture and maintenance of cell morphology

To investigate BbbF, we created the conditional mutant strain CNTs191 by placing the native *bbbF* (*bb0538*) gene under the control of the synthetic IPTG-inducible promoter P*_pQE30_* (108) (Fig. S5). Like strains VCbb08 and VCbb07, we derived CNTs191 from the LacI-producing parent strain B31-68-LS (Fig. S5). CNTs191 exhibited a profound growth defect upon withdrawal of IPTG from the culture medium (Fig. 1G). By 48 h of *bbbF* expression knockdown, the cells also accumulated severe morphological defects that included membrane blebbing (Fig. 1B). Within 24 h of IPTG withdrawal, transcript levels for *bbbF* and the downstream gene *bb0537* were reduced by 98% and 83%, respectively (Fig. 1H), suggesting that knockdown of *bbbF* expression had polar effects. Meanwhile, expression of the upstream gene *bb0539* (Fig. S2A) was unaffected by IPTG withdrawal (Fig. 1H). Moreover, constitutively expressing a second *bbbF* copy from a shuttle vector (see strain CNTs198, Fig. S5) fully complemented the growth and cell morphology defects caused by *bbbF* (and *bb0537*) expression knockdown (Fig. 1B and I). These results indicate that, like *bbbG*, *bbbF* (but not *bb0537*) is also essential for cell replication and morphology maintenance in *B. burgdorferi*.

### Fluorescent protein-tagged BbbF and BbbG localize to known zones of *B. burgdorferi* cell growth

**Fig 2:**
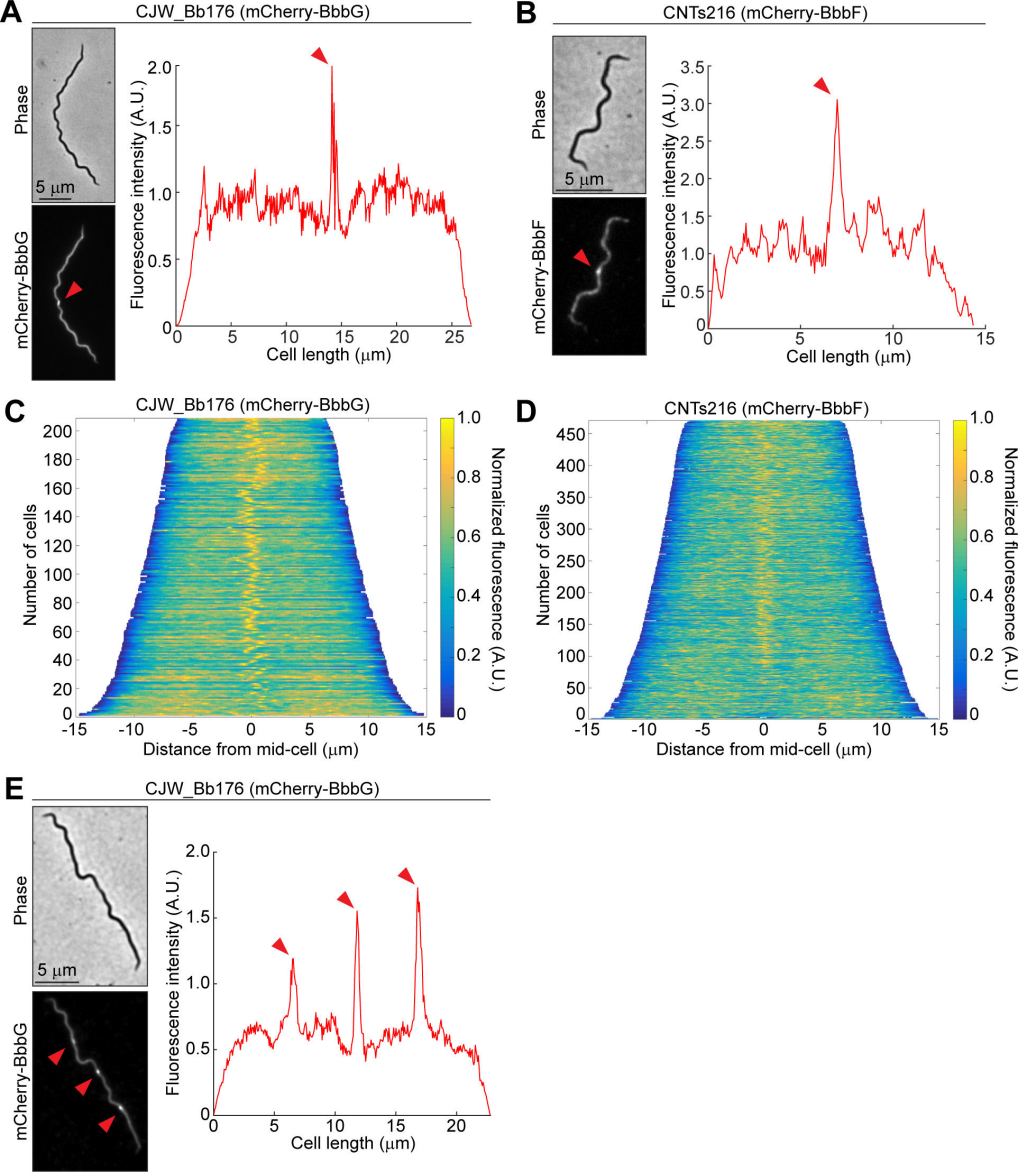
Fluorescence localization in strains expressing mCherry-tagged bactofilins. (**A**) Phase-contrast and fluorescence micrographs (left), and fluorescence intensity profile (right) of a cell of strain CJW_Bb176 expressing *mcherry^Bb^-bbbG*. Mid-cell signal accumulation is indicated by a red arrowhead. A.U., arbitrary units. (**B**) Same as in panel A but for a cell of strain CNTs216 expressing *mcherry^Bb^-bbbF*. (**C**) Demograph analysis of a population of 209 cells imaged from an exponentially growing culture of strain CJW_Bb176. Cells are arrayed from the shortest at the top to the longest at the bottom and are aligned along their mid-cell positions. The color of each line reflects the distribution of the mCherry signal along the length of a single cell, according to the color scale at the right. A.U., arbitrary units. (**D**) Same as in panel C but for a population of 471 cells from strain CNTs216. A.U., arbitrary units. (**E**) Same as in panel A but for a cell of CJW_Bb176 with additional fluorescence accumulation at one-quarter and three-quarter locations along the cell length, as indicated by red arrowheads.

To further investigate BbbF and BbbG, we determined the subcellular localization of mCherry-BbbF and mCherry-BbbG, each produced from a shuttle vector (see strains CNTs216 and CJW_Bb176 in Fig. S5). The fluorescent signal in both mCherry-BbbG- and mCherry-BbbF-producing cells concentrated at mid-cell (Fig. 2A and B). Demograph analyses of populations of cells confirmed mid-cell accumulation of the mCherry signal (Fig. 2C and D). For mCherry-BbbF, mid-cell localization was clearest in spirochetes belonging to the middle ~60% of the cell length distribution (Fig. 2D). In some of the longer cells, we also observed mCherry-BbbG signal accumulation at the one-quarter and three-quarter positions along the cell length (Fig. 2C and E), in a pattern reminiscent of the subcellular distribution of new peptidoglycan insertion sites (97). Western blotting of cell lysates obtained from these strains with an anti-mCherry antibody revealed both full-length mCherry-bactofilin fusions (mCherry-BbbF: expected ~43 kDa; mCherry-BbbG: expected ~48 kDa), as well as a smaller protein similar in size to free mCherry (~27 kDa), most prominent in lysates from cells expressing mCherry-BbbG (Fig. S7A). This result suggests that the mCherry-BbbG and perhaps mCherry-BbbF constructs undergo processing within the bactofilin N-terminal tail, a phenomenon previously documented for *Myxococcus xanthus* and *Proteus mirabilis* bactofilins (92, 111), or within the mCherry-bactofilin linker. However, as neither free mCherry nor mCherry fused to BbbG N-terminal fragments exhibited mid-cell accumulation above the cellular background (Fig. S8A through I), we are confident that the full-length mCherry-bactofilin fusions generated the observed patterns of signal localization (Fig. 2).

### BbbG and BbbF spatially organize peptidoglycan insertion in *B. burgdorferi*

**Fig 3:**
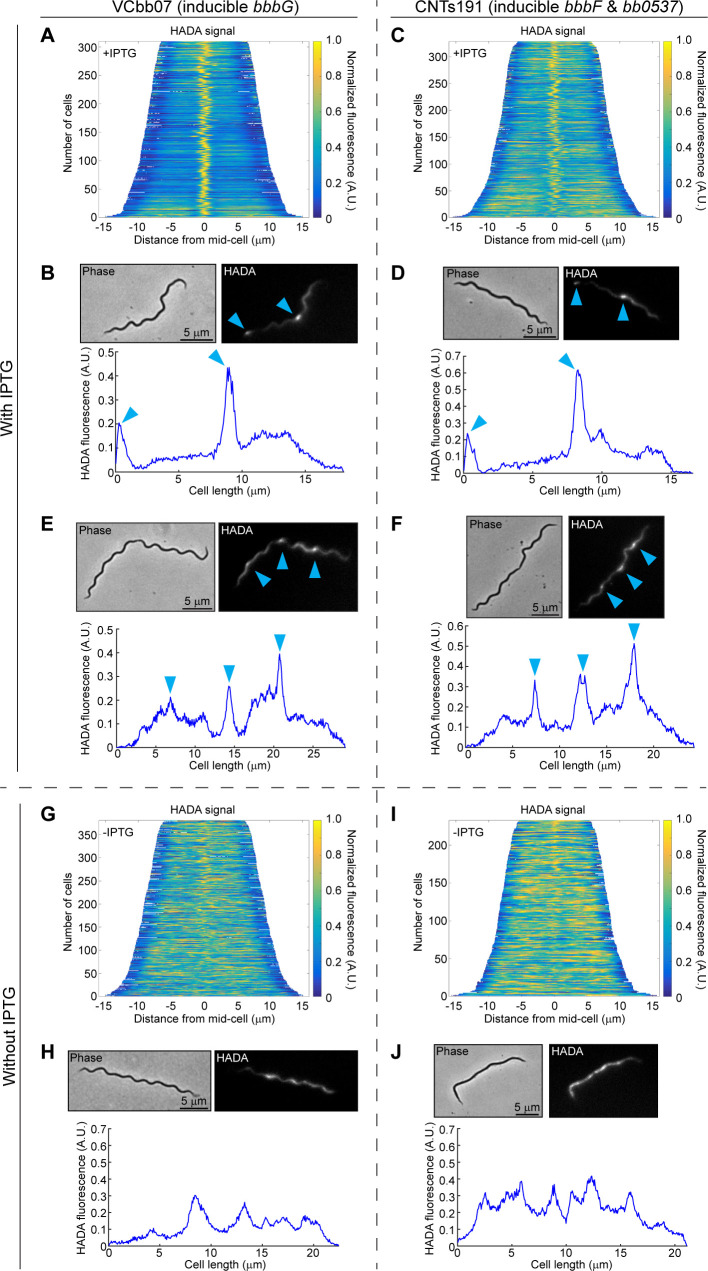
Effects of bactofilin expression knockdown on the localization of new peptidoglycan insertion. (**A**) Demograph of a population of 310 cells of strain VCbb08 (carrying IPTG-inducible *bbbG*) grown in the presence of 100 μM IPTG and pulse-labeled with HADA for 60 min. Cells are arrayed from the shortest at the top to the longest at the bottom and are aligned along their mid-cell positions. The color of each line reflects the distribution of the HADA signal along the cell length in a single cell, according to the color scale at the right. A.U., arbitrary units. (**B**) Phase-contrast and fluorescence micrographs (top), and fluorescence intensity profile (bottom) of a cell from panel A. Mid-cell and polar accumulation are indicated by cyan arrowheads. A.U., arbitrary units. (**C**) Same as in panel A but for a population of 329 cells of strain CNTs191 (carrying IPTG-inducible *bbbF* and *bb0537*) grown in the presence of 100 μM IPTG and pulse-labeled with HADA for 60 min. (**D**) Phase-contrast and fluorescence micrographs (top), and fluorescence intensity profile (bottom) of a cell from panel C. Mid-cell and polar accumulation are indicated by cyan arrowheads. A.U., arbitrary units. (**E**) Same as in panel B for an additional cell from panel A. Signal accumulation at mid-cell, one-quarter, and three-quarter locations along the cell length is indicated by cyan arrowheads. (**F**) Same as in panel D for an additional cell from panel C. Signal accumulation at mid-cell, one-quarter, and three-quarter locations along the cell length is indicated by cyan arrowheads. (**G**) Same as in panel A but for a population of 381 cells of strain VCbb08 grown in the absence of IPTG for 24 h, then pulse-labeled with HADA for 60 min. (**H**) Phase-contrast and fluorescence micrographs (top), and fluorescence intensity profile (bottom) of a cell from panel G. A.U., arbitrary units. (**I**) Same as in panel A but for a population of 233 cells of strain CNTs191 grown in the absence of IPTG for 24 h, then pulse-labeled with HADA for 60 min. (**J**) Phase-contrast and fluorescence micrographs (top), and fluorescence intensity profile (bottom) of a cell from panel I. A.U., arbitrary units.

Since mCherry-tagged BbbG and BbbF accumulated at subcellular locations where growth occurs (Fig. 2), we tested whether these bactofilins direct peptidoglycan insertion. To this end, we pulse-labeled cells of the *bbbG* and *bbbF* expression knockdown strains VCbb07 and CNTs191 (Fig. S5), respectively, with the fluorescent D-amino acid analog 7-hydroxycoumarin-amino-D-alanine (HADA), which labels new sites of peptidoglycan insertion in various bacteria, including *B. burgdorferi* (97, 98, 112). When grown in the presence of IPTG to maintain bactofilin expression, both strains accumulated HADA signal predominantly at mid-cell (Fig. 3A through D), while some longer cells also accumulated HADA signal at one-quarter and three-quarter cell positions (Fig. 3E and F). Additionally, we observed unipolar HADA signal accumulation in some, mostly shorter cells (Fig. 3B and D), consistent with septal synthesis and new pole formation during the HADA pulse, as previously reported (97).

In these strains, removal of IPTG 24 h prior to HADA labeling yielded cells with patchy fluorescence along most of the cell length, except at the poles (Fig. 3G through J). These patches of HADA signal were heterogeneous in size and intensity and were observed in both shorter and longer cells (Fig. 3G through J). Some mid-cell signal was still observed (Fig. 3G and I), likely due to incomplete depletion of the bactofilins over approximately three generations of IPTG-free growth. The amount of HADA fluorescence signal was not affected by *bbbG* expression knockdown in strain VCbb07 (Fig. S9), indicating that HADA signal delocalization in this strain was not caused by decreased uptake. Moreover, cells of strain CNTs191 in which we knocked down expression of *bbbF* displayed the same levels of HADA uptake as cells of strain VCbb07 grown with or without IPTG (Fig. S9), again indicating that HADA delocalization in this strain was not caused by decreased uptake. We did, however, observe increased HADA fluorescence in *bbbF*-expressing cells of strain CNTs191 (Fig. S9), which may suggest that induction of *bbbF* expression stimulates peptidoglycan synthesis. Since prolonged bactofilin expression knockdown caused membrane blebbing (Fig. 1B and C), the resulting cells were not amenable to washing or generating the cell outlines required for quantitative image analysis. Consequently, we could not reliably assess the effects of extended bactofilin knockdown on peptidoglycan insertion. Nevertheless, our results implicate BbbG and BbbF in the localization of new peptidoglycan insertion in *B. burgdorferi*.

### Bactofilins co-localize with newly inserted peptidoglycan

To further investigate the role bactofilins play in *B. burgdorferi* peptidoglycan insertion, we generated strains CNTs217, which constitutively expresses *mcherry^Bb^-bbbF* and inducibly expresses native *bbbF* and *bb0537*, and strain CJW_Bb279, which constitutively expresses *mcherry^Bb^-bbbG* and inducibly expresses native *bbbG* and *bb0244*, respectively (Fig. S5). Culture growth (Fig. S10A and B), spirochetal morphology (Fig. S10C), mCherry-bactofilin localization (Fig. S10D through G), and HADA insertion patterns (Fig. S10D through G) were all unaffected by downregulation of native bactofilin gene expression through growth in IPTG-free media. These observations indicate that mCherry-BbbF and mCherry-BbbG are functional.

**Fig 4:**
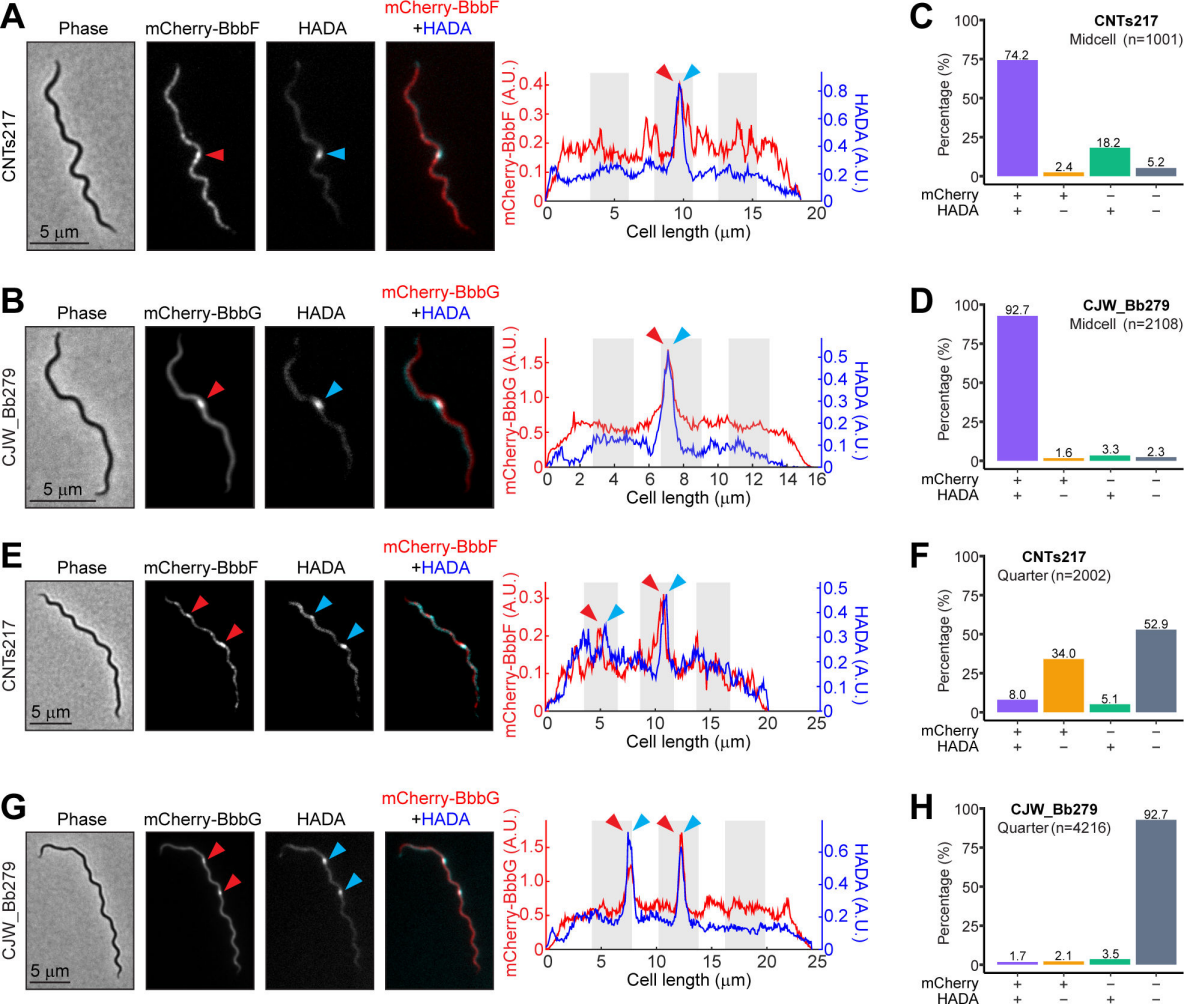
Colocalization of mCherry-BbbF and mCherry-BbbG with peptidoglycan insertion. (**A**) Phase-contrast and fluorescence micrographs (left), and fluorescence intensity profile (right) for a cell of strain CNTs217, which expresses *mcherry^Bb^-bbbF*, grown with 100 µM IPTG and labeled with HADA. From left to right, the images are as follows: phase-contrast micrograph; fluorescence micrograph of mCherry-BbbF signal; fluorescence micrograph of HADA signal; and overlaid image of the mCherry-BbbF signal (in red) with the HADA signal (in cyan). Mid-cell mCherry-BbbF and HADA peaks are indicated by red and cyan arrowheads, respectively. Right, fluorescence intensity profile along the cell length. The mCherry-BbbF signal is in red, while the HADA signal is in blue. A.U., arbitrary units. (**B**) Phase-contrast and fluorescence micrographs (left), and fluorescence intensity profile (right) for a cell of strain CJW_Bb279, which expresses *mcherry^Bb^-bbbG*, grown with 100 µM IPTG and labeled with HADA. From left to right, the images are as follows: phase-contrast micrograph; fluorescence micrograph of mCherry-BbbG signal; fluorescence micrograph of HADA signal; and overlaid image of the mCherry-BbbG signal (in red) with the HADA signal (in cyan). Mid-cell mCherry-BbbG and HADA peaks are indicated by red and cyan arrowheads, respectively. Right, fluorescence intensity profile along the cell length. The mCherry-BbbG signal is in red, while the HADA signal is in blue. A.U., arbitrary units. (**C**) Bar graph quantifying the presence of mid-cell mCherry-BbbF and/or HADA peaks in a population of 1,001 cells of strain CNTs217 grown with 100 µM IPTG and labeled with HADA. Mid-cell signal peaks were defined using a fluorescent spot finder algorithm restricted to a cell region centered at mid-cell and corresponding to 15% of cell length. From left to right, the bars indicate the fraction of mid-cell regions containing both an mCherry-BbbF and a HADA peak, only an mCherry-BbbF peak, only a HADA peak, and no peak in either of the signals, respectively. (**D**) Bar graph quantifying the presence of mid-cell mCherry-BbbG and/or HADA peaks in a population of 2,108 cells of strain CJW_Bb279 grown with 100 µM IPTG and labeled with HADA. Mid-cell signal peaks were defined using a fluorescent spot finder algorithm restricted to a cell region centered at mid-cell and corresponding to 15% of cell length. From left to right, the bars indicate the fraction of mid-cell regions containing both an mCherry-BbbG and a HADA peak, only an mCherry-BbbG peak, only a HADA peak, and no peak in either of the signals, respectively. (**E**) Same as in panel A but for a different cell of strain CNTs217 grown with 100 µM IPTG and labeled with HADA. Mid-cell and one-quarter-cell mCherry-BbbF and HADA peaks are indicated by red and cyan arrowheads, respectively. (**F**) Same as in panel C but quantifying the presence of mCherry-BbbF and/or HADA peaks in the one-quarter and three-quarter cell regions (*n* = 2,002 quarter-cell regions in 1,001 cells). Quarter-cell signal peaks were defined using a fluorescent spot finder algorithm restricted to the cell regions centered at the one-quarter and three-quarter cell positions and corresponding to 15% of cell length. (**G**) Same as in panel B but for a different cell of strain CJW_Bb279 grown with 100 µM IPTG and labeled with HADA. Mid-cell and one-quarter-cell mCherry-BbbG and HADA peaks are indicated by red and cyan arrowheads, respectively. (**H**) Same as in panel **D** Dut quantifying the presence of mCherry-BbbG and/or HADA peaks in the one-quarter and three-quarter cell regions (*n* = 4,216 quarter-cell regions in 2,108 cells). Quarter-cell signal peaks were defined using a fluorescent spot finder algorithm restricted to the cell regions centered at the one-quarter and three-quarter cell positions and corresponding to 15% of cell length. (**A**,** B, E, and G**) The cell regions centered at mid-cell, one-quarter, and three-quarter positions, and each corresponding to 15% of the cell’s length, are highlighted in gray in the intensity profile graphs.

We next assessed whether the tagged bactofilins localized to the known zones of peptidoglycan insertion by performing spot detection in cells of strains CNTs217 and CJW_Bb279 grown with IPTG and pulse-labeled with HADA. We restricted our analysis to windows representing 15% of each cell’s length and centered around mid-cell, as well as the one-quarter and three-quarter positions, and marked by gray shading in fluorescence intensity profiles in Fig. 4. Consistent with our demograph analyses (Fig. S10D through G), we found that mid-cell HADA peaks colocalized with mCherry-BbbF (Fig. 4A) or mCherry-BbbG (Fig. 4B) in 74.2% (743/1,001) and 92.7% (1,955/2,108) of all analyzed cells, respectively (Fig. 4C and D). Within the one-quarter and three-quarter cell regions, mCherry-BbbF and HADA peaks co-occurred, but were not always colocalized, in 8.0% (160/2002, Fig. 4E and F) of cases, while mCherry-BbbG and HADA colocalized in 1.7% (72/4216) of cases (Fig. 4G and H), respectively. The overall pattern of mCherry-bactofilin and HADA signal co-accumulation was unchanged when we grew these strains without IPTG (Fig. S10H through K). As further expounded in the discussion, these findings are consistent with our hypothesis that bactofilins BbbF and BbbG direct the insertion of new peptidoglycan.

### Bactofilin accumulation precedes peptidoglycan insertion at new growth zones

**Fig 5:**
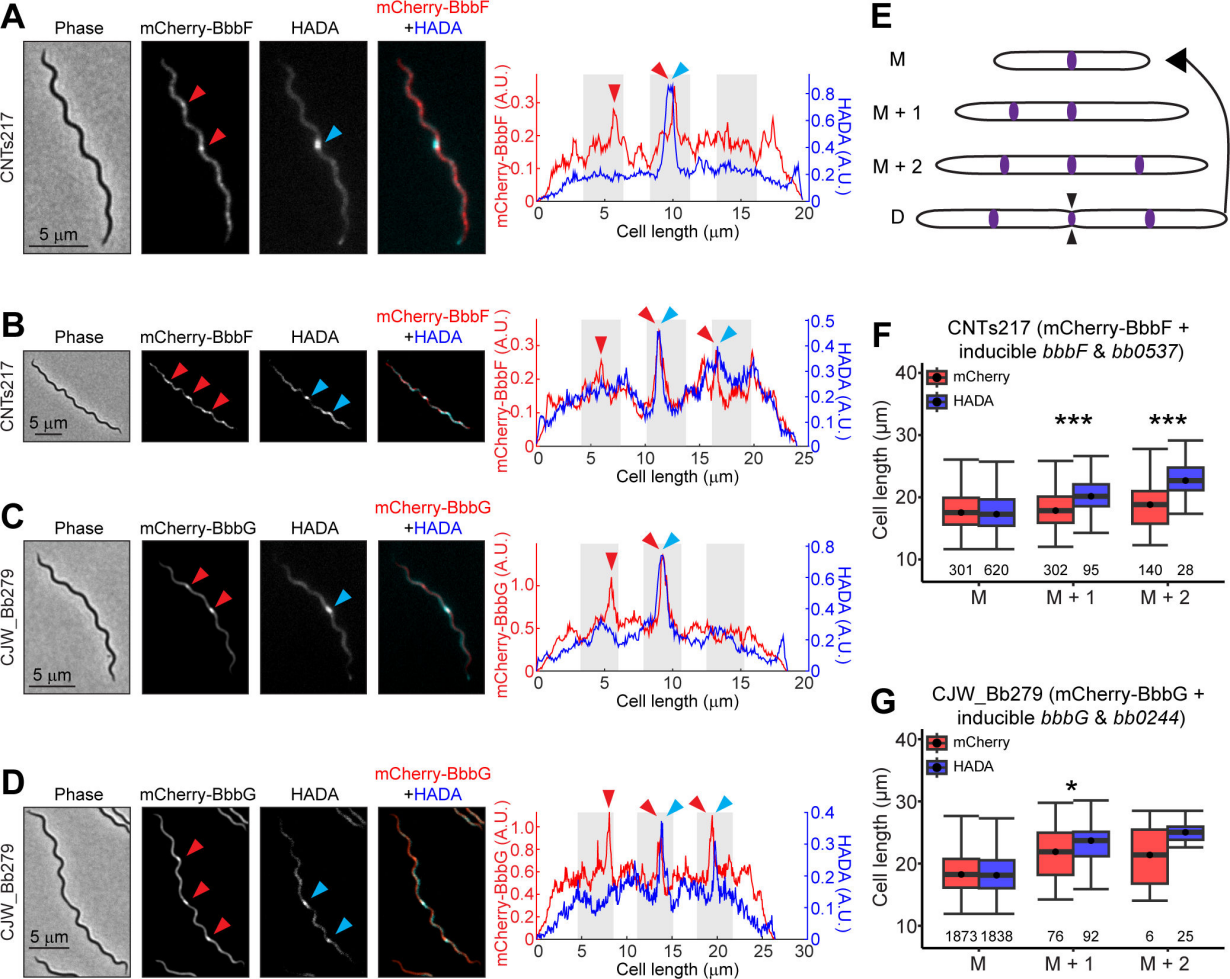
Evidence of bactofilin localization at quarter-cell positions prior to peptidoglycan insertion. (**A and B**) Phase-contrast and fluorescence micrographs (left), and fluorescence intensity profiles (right) for cells of strain CNTs217, which expresses *mcherry^Bb^-bbbF*, grown with 100 µM IPTG and labeled with HADA. From left to right, the images are as follows: phase-contrast micrograph; fluorescence micrograph of mCherry-BbbF signal; fluorescence micrograph of HADA signal; and overlaid image of the mCherry-BbbF signal (in red) with the HADA signal (in cyan). Mid-cell and quarter-cell mCherry-BbbF and HADA peaks are indicated by red and cyan arrowheads, respectively. Right, fluorescence intensity profiles along the cell length. The mCherry-BbbF signal is in red, while the HADA signal is in blue. A.U., arbitrary units. The panels show mCherry-BbbF accumulation in the absence of a HADA peak at the one-quarter cell length region in the absence (**A**) or presence (**B**) of mCherry-BbbF and HADA peaks at the three-quarter cell length region. (**C and D**) Phase-contrast and fluorescence micrographs (left), and fluorescence intensity profiles (right) for cells of strain CJW_Bb279, which expresses *mcherry^Bb^-bbbG*, grown with 100 µM IPTG and labeled with HADA. From left to right, the images are as follows: phase-contrast micrograph; fluorescence micrograph of mCherry-BbbG signal; fluorescence micrograph of HADA signal; and overlaid image of the mCherry-BbbG signal (in red) with the HADA signal (in cyan). Mid-cell and quarter-cell mCherry-BbbG and HADA peaks are indicated by red and cyan arrowheads, respectively. Right, fluorescence intensity profiles along the cell length. The mCherry-BbbG signal is in red, while the HADA signal is in blue. A.U., arbitrary units. The panels show mCherry-BbbG accumulation in the absence of a HADA peak at the one-quarter cell length region in the absence (C) or presence (D) of mCherry-BbbG and HADA peaks at the three-quarter cell length region. (**A–D**) The cell regions centered at mid-cell, one-quarter, and three-quarter positions, each corresponding to 15% of the cell’s length, are highlighted in gray in the intensity profile graphs. (**E**) Schematic representation of *B. burgdorferi* growth highlighting asynchronous bactofilin accumulation or peptidoglycan insertion at the one-quarter and three-quarter cell length regions. Classification of the cells by growth stages into mid-cell only (M), mid-cell and one-quarter (M + 1), mid-cell, one-quarter and three-quarter (M + 2), and divisional (**D**) categories is highlighted at the left. Since we currently lack the ability to distinguish between old-pole and new-pole halves of a *B. burgdorferi* cell, the one-quarter and three-quarter positions are considered equivalent for the purpose of our analyses. (**F**) Box plots depicting the length distributions of cells of strain CNTs217 (*n* = 743 from two independent experiments) grown with 100 µM IPTG and labeled with HADA. Cells were grouped based on the mCherry-BbbF signal (in red) or the HADA signal (in blue) into the M, M + 1, and M + 2 classes as defined in panel E. Panels A and B of Fig. S11 contain the same analysis performed on the independent replicates. (**G**) Box plots depicting the length distributions of cells of strain CJW_Bb279 (*n* = 1,955 from two independent experiments) grown with 100 µM IPTG and labeled with HADA. Cells were grouped based on the mCherry-BbbG signal (in red) or the HADA signal (in blue) into the M, M + 1, and M + 2 classes as defined in panel E. Panels C and D of Fig. S11 contain the same analysis performed on the independent replicates. (**F and G**) For each signal and class, the numbers of analyzed cells are shown above the x-axis. Boxes represent the interquartile ranges of cell lengths with the median as the midline; tails represent the 2.5 and 97.5 percentiles. The two-tailed, paired Wilcoxon test was used to measure significance of the differences in median cell length within each growth stage between the grouping based on the mCherry-bactofilin signal and that based on the HADA signal. *P*-values are shown above the box plots: ***, *P* < 0.0001; **, *P* < 0.001; *, *P* < 0.01.

Within the same image sets, we also identified cells in which the tagged bactofilins formed peaks in the absence of zonal HADA accumulation (Fig. 5A through D). This occurred in 34.0% of all quarter-cell regions for mCherry-BbbF (Fig. 4F and Fig. 5A and B) and 2.1% for mCherry-BbbG (Fig. 4H and Fig. 5C and D). Since the HADA signal represents peptidoglycan inserted during the 1-hour pulse preceding imaging, while the mCherry signal indicates the localization of the tagged bactofilins at the time of imaging, the presence of bactofilin peaks and the absence of peptidoglycan peaks suggested that the bactofilins can arrive at the one-quarter and three-quarter cell locations before peptidoglycan insertion begins. Moreover, in addition to cells that displayed bactofilin or HADA signal accumulation at mid-cell, or at mid-cell and both one-quarter and three-quarter cell positions (Fig. 2, Fig. 3A through F and Fig. 4A and B), we also observed cells with signal accumulation at mid-cell and one-quarter cell positions, without accumulation at the three-quarter cell position (Fig. 4E and G and Fig. 5A through D). This suggests that the one-quarter and three-quarter zones of *B. burgdorferi* cell growth can develop asynchronously (Fig. 5E), a phenomenon previously implied but not investigated in detail (97, 98, 100). We exploited this observation and the known correlation between bacterial cell length and cell cycle progression to gain a population-level understanding of the temporal order in which bactofilin recruitment and zonal peptidoglycan synthesis occur at nascent growth zones. For both bactofilin and HADA signals, we grouped the cells into categories with accumulation at mid-cell only (M), mid-cell and a one-quarter position (M + 1), and mid-cell, one-quarter and three-quarter positions (M + 2) (Fig. 5E). Cells that did not have a detectable signal peak at mid-cell for either signal were excluded from this analysis (Table S2). As expected, increasing cell length positively correlated with increasing number of either HADA or mCherry-bactofilin foci (Tables S2 and S3). We next compared cell length distributions within each category (Fig. 5F and G, Fig. S11A through D and Table S4). Cells sorted into the M class based on their mCherry-bactofilin signal had lengths statistically indistinguishable from those of cells sorted into the M class based on their HADA signal (Fig. 5F and G and Table S5). In contrast, cells sorted into the M + 1 class using either the mCherry-BbbG or mCherry-BbbF signals were significantly shorter than cells sorted into the M + 1 class by their HADA signal (Fig. 5F and G and Table S5). Similarly, cells sorted into the M + 2 class by their mCherry-BbbF signal were significantly shorter than cells sorted into the M + 2 class by their HADA signal (Fig. 5F and Table S5). The same comparison for mCherry-BbbG found no statistically significant difference, possibly due to the low cell numbers in the M + 2 classes derived from this population (Fig. 5G and Table S5). Altogether, this analysis indicates that the tagged bactofilins localize to the one-quarter and three-quarter cell positions in shorter cells than those in which zonal peptidoglycan insertion at the same locations becomes detectable. This implies that the bactofilins localize to new hotspots of cell wall growth prior to new peptidoglycan insertion at these locations.

### *B. burgdorferi* FtsA localization is unaffected by bactofilin depletion

**Fig 6:**
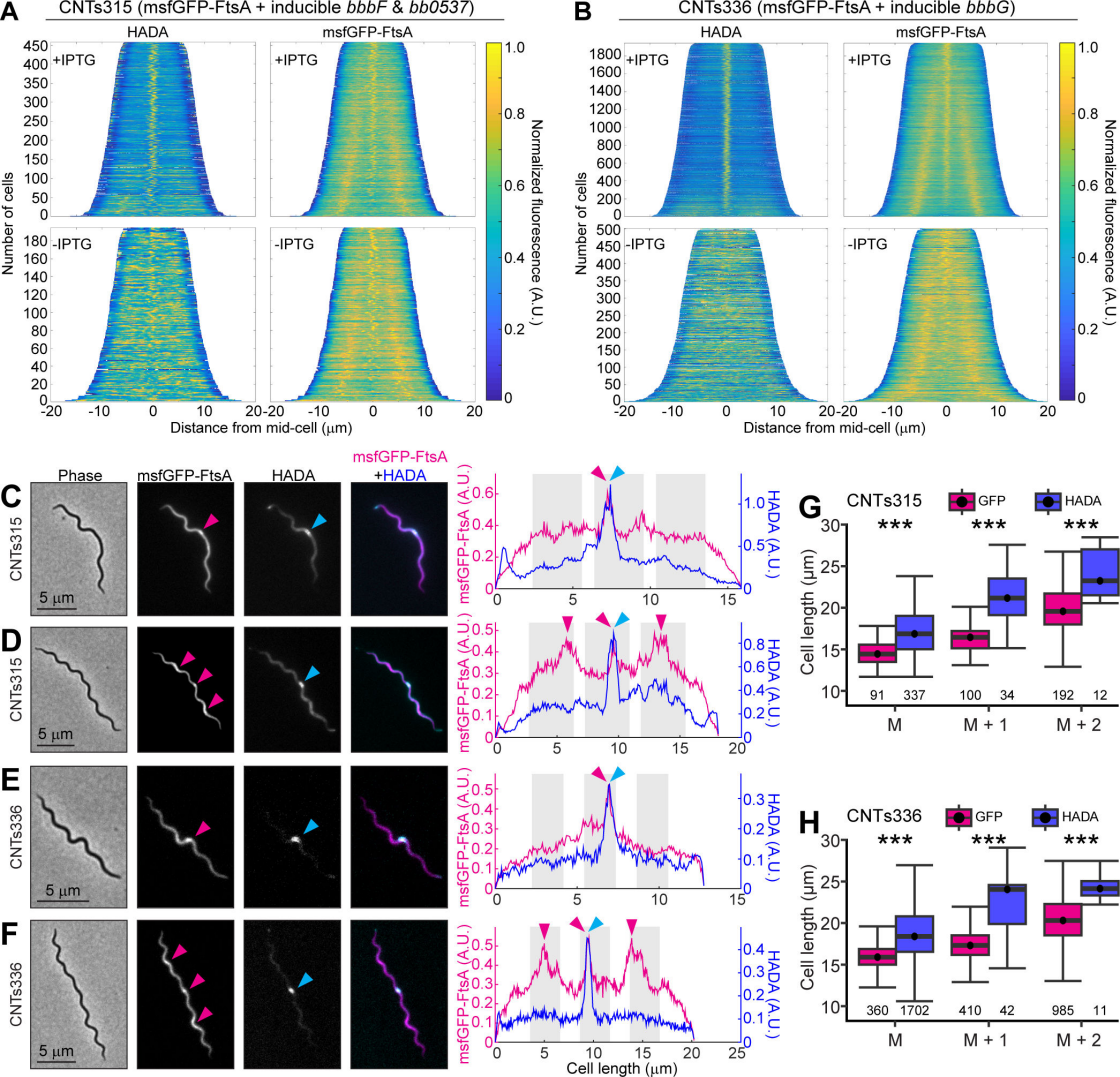
Effects of bactofilin expression knockdown on msfGFP-FtsA localization. (**A**) Demographs of populations of cells of strain CNTs315, which constitutively expresses *msfgfp^Bb^-ftsA* and inducibly expresses *bbbF* and *bb0537,* grown with 100 μM IPTG (top, *n* = 458 cells) or grown in the absence of IPTG for 24 h (bottom, *n* = 198 cells). (**B**) Demographs of populations of cells of strain CNTs336, which constitutively expresses *msfgfp^Bb^-ftsA* and inducibly-expresses *bbbG* and *bb0244,* grown with 100 μM IPTG (top, *n* = 1,947 cells) or grown in the absence of IPTG for 24 h (bottom, *n* = 501 cells). (**A and B**) The cells were labeled with HADA for 60 min prior to imaging. Demographs of the HADA signal are on the left, while those of the msfGFP-FtsA signal are on the right. The cells are arrayed from the shortest at the top to the longest at the bottom and are aligned along their mid-cell positions. The color of each line reflects the distribution of the fluorescent signal along the cell length, according to the color scale at the right. A.U., arbitrary units. (**C and D**) Phase-contrast and fluorescence micrographs (left), and fluorescence intensity profiles (right) for cells of strain CNTs315 grown with 100 µM IPTG and labeled with HADA. (**E and F**) Phase-contrast and fluorescence micrographs (left), and fluorescence intensity profiles (right) for cells of strain CNTs336 grown with 100 µM IPTG and labeled with HADA. (**C–F**) From left to right, the images are as follows: phase-contrast micrograph; fluorescence micrograph of msfGFP-FtsA signal; fluorescence micrograph of HADA signal; and overlaid image of the msfGFP-FtsA signal (in magenta) with the HADA signal (in cyan). Right, fluorescence intensity profiles along the cell length. The msfGFP-FtsA signal is in magenta, while the HADA signal is in blue. A.U., arbitrary units. The cell regions centered at mid-cell, one-quarter, and three-quarter positions, and each corresponding to 20% of the cell’s length, are highlighted in gray in the intensity profile graphs. Mid-cell and quarter-cell msfGFP-FtsA and HADA peaks are indicated by magenta and cyan arrowheads, respectively. Cells in panels D and F show msfGFP-FtsA signal accumulation within the one-quarter and three-quarter cell length regions in the absence of HADA signal accumulation. (**G**) Box plots depicting the length distributions of cells of strain CNTs315 (*n* = 383) grown with 100 µM IPTG and labeled with HADA. Cells were grouped based on the msfGFP-FtsA signal (in magenta) or the HADA signal (in blue) into the M, M + 1, and M + 2 classes as defined in Fig. 5E. (**H**) Same as in panel G for cells of strain CNTs336 (*n* = 1,755) grown with 100 µM IPTG and labeled with HADA. (**G and H**) For each signal and class, the numbers of analyzed cells are shown above the x-axis. Boxes represent the interquartile ranges of cell lengths with the median as the midline; tails represent the 2.5 and 97.5 percentiles. The two-tailed, paired Wilcoxon test was used to measure the significance of the differences in median cell length within each growth stage between the grouping based on the msfGFP-FtsA signal and that based on the HADA signal. *P*-values are shown above the box plots: ***, *P* < 0.0001; **, *P* < 0.001; *, *P* < 0.01.

Recently, *B. burgdorferi* FtsA was reported to localize to new zones of cell growth early in the cell cycle and to precede peptidoglycan insertion at these locations (100). To assess connections between FtsA and bactofilins in directing new peptidoglycan insertion, we introduced *msfgfp^Bb^-ftsA* into our bactofilin knockdown strains VCbb07 and CNTs191, yielding strains CNTs336 and CNTs315, respectively (Fig. S5). When grown with IPTG, peptidoglycan insertion occurred as expected, predominantly at mid-cell and with longer cells displaying additional insertion hotspots at the one-quarter and/or three-quarter positions (Fig. 6A and B, top demographs, Fig. 6C through F). In these cells, msfGFP-FtsA also localized at mid-cell (Fig. 6A and B, top demographs, and Fig. 6C and E), with additional one-quarter and three-quarter accumulation zones becoming evident early in the cell cycle and often occurring before peptidoglycan insertion notably increased at those locations (Fig. 6A and B, top demographs, and Fig. 6D and F). We again grouped the cells into M, M + 1, and M + 2 localization classes (Fig. 5E and Table S3) based either on their msfGFP-FtsA or HADA signals. For each class, cells grouped by the msfGFP-FtsA signal were significantly shorter than cells grouped by the HADA signal (Fig. 6G and H and Table S5), demonstrating that msfGFP-FtsA accumulates at new zones of cell growth before zonal peptidoglycan insertion occurs, in agreement with the previous report (100). Importantly, knockdown of either *bbbF* or *bbbG* expression in these strains (by growing them in the absence of IPTG for 24 h) delocalized peptidoglycan insertion but left msfGFP-FtsA localization largely unaffected (Fig. 6A and B, bottom demographs), indicating that the bactofilins direct peptidoglycan insertion but not FtsA localization.

### FtsA precedes bactofilin recruitment to new zones of peptidoglycan insertion

**Fig 7:**
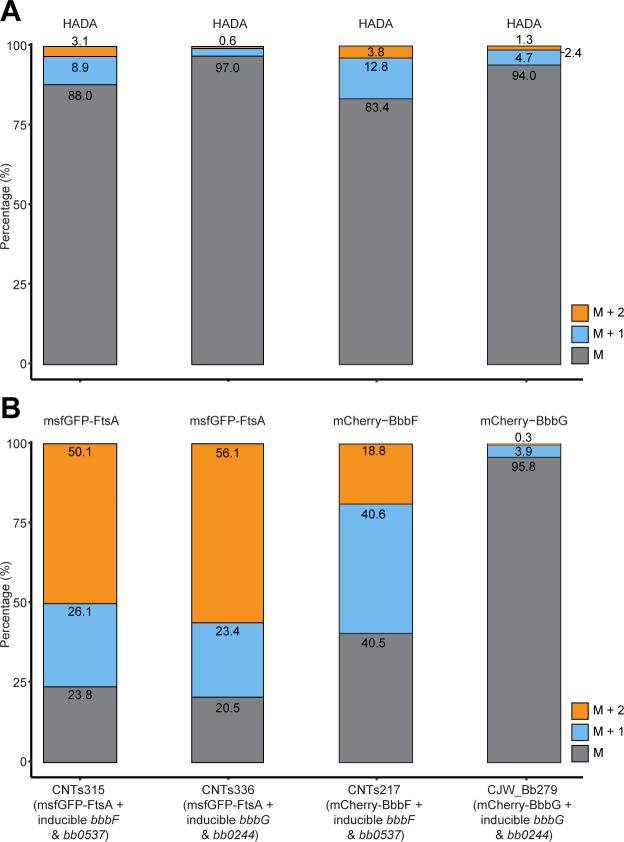
Population-level comparison of the prevalence of msfGFP-FtsA, mCherry-BbbF, mCherry-BbbG, and peptidoglycan insertion at subcellular locations of zonal growth. (**A**) Abundance of peptidoglycan insertion (HADA signal) classes M, M + 1, and M + 2 (as defined in Fig. 5E) in cell populations of strains (from left to right): CNTs315 (*n* = 383), CNTs336 (*n* = 1,755), CNTs217 (*n* = 743), and CJW_Bb279 (*n* = 1,955), respectively, grown with 100 µM IPTG. Numbers inside the bars indicate the percent of the population belonging to each class. (**B**) Same as in panel A but when classes M, M + 1, and M + 2 were defined based on the msfGFP-FtsA signal (for strains CNTs315 and CNTs336), mCherry-BbbF signal (for strain CNTs217), and mCherry-BbbG signal (for strain CJW_Bb279), respectively.

Our demograph-based visualization of msfGFP-FtsA, mCherry-BbbF, mCherry-BbbG, and HADA-labeled new peptidoglycan insertion (Fig. 2C and D, Fig. 3A and B and Fig. 6A and B) implied that msfGFP-FtsA arrives early at these locations, before the bactofilins. To gain further insight into the order of events occurring during the cell cycle, we compared the relative abundance of cells belonging to the M, M + 1, and M + 2 localization classes for each signal. While we performed these analyses on different strains, each experiment included HADA labeling, which allowed us to compare the data across strains. For all strains, most cells in the population (class M, range: 83.4% to 97%) had one HADA peak at mid-cell, followed by smaller fractions belonging to the M + 1 class (range: 2.4% to 12.8%) and even smaller fractions belonging to the M + 2 class (range: 0.6% to 3.8%) (Fig. 7A). This suggests that insertion of peptidoglycan at one-quarter and three-quarter cell positions occurs late in the cell cycle, in agreement with previous findings (97). In contrast (see Fig. 7B), in the two msfGFP-FtsA-producing strains, the M class was the least represented (range: 20.5% to 23.8%), followed by the M + 1 class (range: 23.4% to 26.1%), with the M + 2 class being most abundant (range: 50.1% to 56.1%). mCherry-BbbF-producing cells had a lower (18.8%) abundance of cells belonging to the M + 2 class, with the remaining cells distributed equally among the M and M + 1 classes. Finally, grouping based on the mCherry-BbbG signal revealed that most cells (95.8%) belonged to the M class. This result suggests that BbbF arrives at new zones of cell growth after FtsA, but before BbbG.

**Fig 8:**
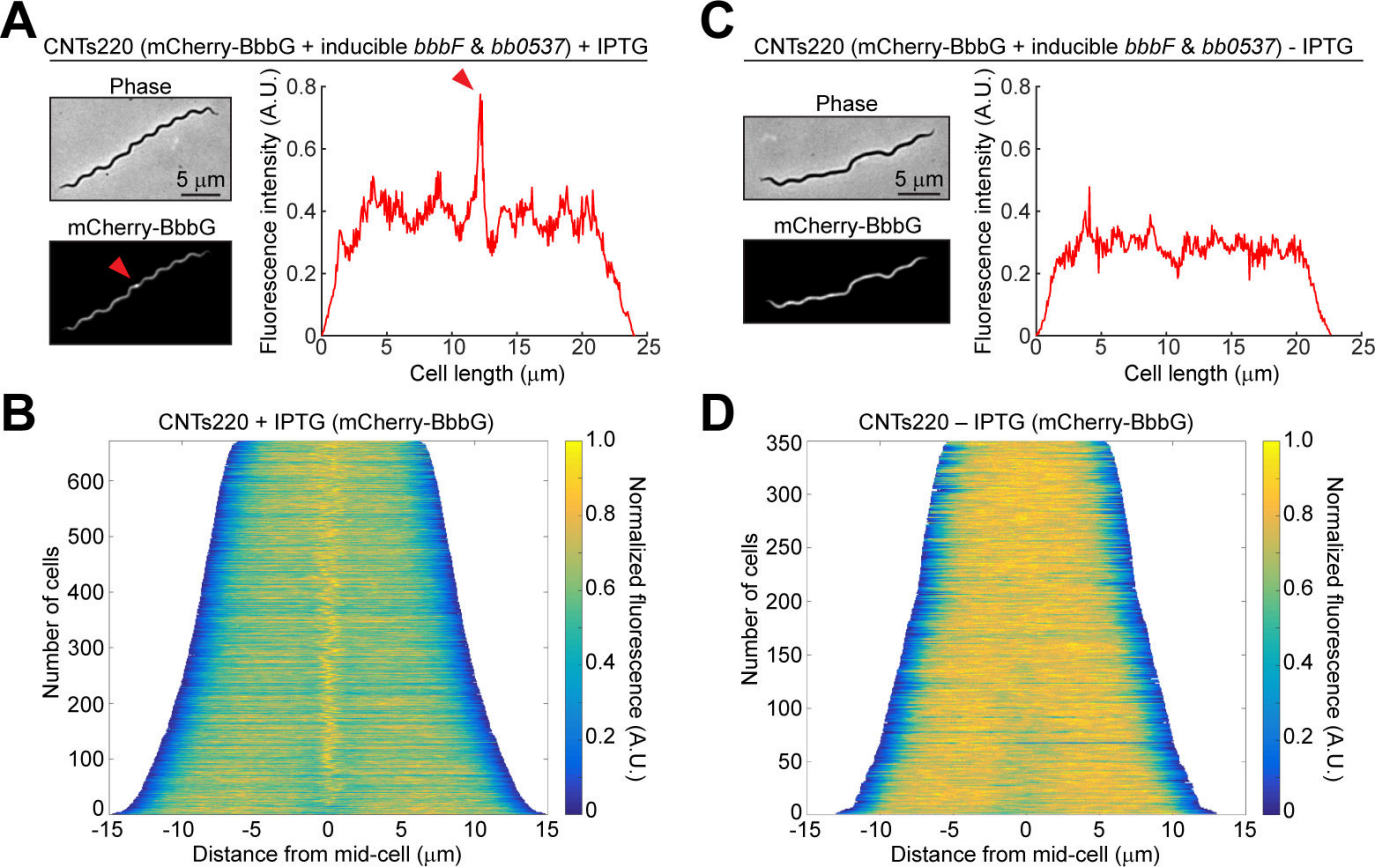
mCherry-BbbG requires *bbbF* expression to localize at mid-cell and quarter-cell positions. (**A**) Phase-contrast and fluorescence micrographs (left), and fluorescence intensity profile (right) of a cell of strain CNTs220 which constitutively expresses *mcherry^Bb^-bbbG* and inducibly expresses *bbbF* and *bb0537,* grown with 1 mM IPTG. Mid-cell accumulation of the mCherry signal is indicated by a red arrowhead. A.U., arbitrary units. (**B**) Demograph of a population of 672 cells of strain CNTs220 grown in the presence of 1 mM IPTG. Cells are arrayed from the shortest at the top to the longest at the bottom and are aligned along their mid-cell positions. The color of each line reflects the distribution of the mCherry signal along the cell length, according to the color scale at the right. A.U., arbitrary units. (**C**) Phase-contrast and fluorescence micrographs (left), and fluorescence intensity profile (right) of a cell of strain CNTs220 grown in the absence of IPTG for 24 h. A.U., arbitrary units. (**D**) Demograph of a population of 350 cells of strain CNTs220 grown in the absence of IPTG for 24 h. Cells are arrayed from the shortest at the top to the longest at the bottom and are aligned along their mid-cell positions. The color of each line reflects the distribution of the mCherry signal along the cell length, according to the color scale at the right. A.U., arbitrary units.

### BbbG localization requires BbbF

Since knockdown of *bbbF* and *bbbG* expression yielded similar phenotypes (Fig. 1 and Fig. 3), but mCherry-BbbF appeared to localize to quarter-cell positions before mCherry-BbbG (Fig. 7B), we hypothesized that the two bactofilins function in the same pathway, with BbbF functioning upstream of BbbG. To test this, we expressed *mcherry^Bb^-bbbG* in the IPTG-inducible *bbbF* and *bb0537* background (strain CNTs220, Fig. S5). This strain exhibited mid-cell accumulation of the mCherry signal when *bbbF* was expressed during growth in the presence of IPTG (Fig. 8A and B). In contrast, knockdown of *bbbF* expression obtained by incubation in IPTG-free media delocalized the mCherry-BbbG signal (Fig. 8C and D) without affecting mCherry-BbbG expression or processing (Fig. S7B). We therefore conclude that BbbF is required for the observed mCherry-BbbG localization to mid-cell and to the one-quarter and three-quarter cell positions.

## DISCUSSION

Here, we report an initial characterization of *B. burgdorferi* bactofilins BbbF and BbbG. Our preliminary investigation of BbbA suggests that this bactofilin carries out functions distinct from those of BbbF and BbbG, which is why we are not reporting our BbbA findings here. We show, however, that BbbF and BbbG are essential for *B. burgdorferi* viability. We were unable to delete the chromosomal copy of *bbbG* without expressing a second copy of the gene *in trans*. Moreover, knockdown of *bbbF* or *bbbG* expression caused growth arrest and disrupted the cells’ typical morphology. These phenotypes were complemented by expression of the corresponding mCherry-tagged bactofilin *in trans*. In contrast, most bactofilins studied to date are not essential for bacterial growth and viability, as shown by the successful generation of corresponding bactofilin gene deletion mutants (75, 79, 82, 86, 87, 89–92, 96). In *Chlamydia trachomatis*, CRISPR interference was used to knock down bactofilin *bacA_CT_* expression and reveal its role in cell size maintenance (85). In *Bacillus subtilis* strain PY79*,* the bactofilin-encoding genes *bacE* (*yhbE*) and *bacF* (*yhbF*) could not be deleted individually but could be deleted together (113), and single gene deletions could be transferred into this background from strain 168 (81).

Our investigation revealed that functional mCherry-tagged versions of BbbF and BbbG localized to the known primary zones of *B. burgdorferi* cell growth. Moreover, we found that knockdown of either *bbbF* or *bbbG* expression delocalized peptidoglycan insertion, including from mid-cell, followed by membrane blebbing (Fig. 1 and Fig. 3). Joint regulation of a cellular process by multiple bactofilins has been previously described in *C. crescentus* (75), *M. xanthus* (82), and *Rhodomicrobium vannielii* (86). While mCherry-BbbF and mCherry-BbbG accumulate at zones of peptidoglycan insertion, the timing of their arrival at these locations differs (Fig. 5H and I). mCherry-BbbF appears to localize in the vicinity of quarter-cell positions before mCherry-BbbG (Fig. 4G and H and Fig. 5F and G) and is required for the localization of BbbG (Fig. 6). It is possible that a dedicated recruitment factor localizes there and then recruits BbbF, similar to how SpmX recruits bactofilin BacA to the site of future stalk synthesis in *Asticaccaulis biprosthecum* (79). In *H. pylori*, bactofilin CcmA is thought to recognize positive Gaussian curvature and direct peptidoglycan insertion along the long helical axis of this bacterium, establishing and maintaining helicity (96). A different mechanism may be at work in *L. biflexa*, where the cell’s helical pitch depends on bactofilin LbbD, as demonstrated by opposing pitch changes caused by *lbbD* overexpression and deletion, respectively (91). In *B. burgdorferi*, the pattern of peptidoglycan insertion is similar in cells with wild-type flat-wave morphology and in cells that grow as straight rods after genetic ablation of the flagella through deletion of the flagellin gene *flaB* (50, 97). This implies that BbbF does not rely on membrane curvature to position itself at specified locations along the length of the cell. The mechanism by which BbbF recruits BbbG also remains unknown, but may involve co-polymerization, lateral association of BbbF and BbbG filaments, or BbbF-dependent maturation of the quarter-cell positions into BbbG recruitment-competent locations.

Recently, msfGFP-FtsA was shown to localize at the known *B. burgdorferi* cell growth zones well before significant zonal peptidoglycan insertion occurs (100). We reproduced this observation but also found that downregulating *bbbF* or *bbbG* expression does not delocalize FtsA (Fig. 6). Moreover, downregulating *ftsI* expression or inhibiting FtsI activity using piperacillin prevents cell division but not cell elongation (99, 100, 114). These findings imply that divisome-dependent peptidoglycan synthesis, while required for septation, may not be needed for zonal cell growth. Indeed, we observed tri-zonal localization of mCherry-BbbG in cells treated with piperacillin (Fig. S12A), and recently reported data (100) show zonal peptidoglycan synthesis in a notable subset of elongated, piperacillin-treated cells. While these observations do not rule out a role for FtsA in directing bactofilin localization at the *B. burgdorferi* growth zones, they suggest that zonal cell growth outside division events is mediated not by the divisome but by other peptidoglycan biosynthetic complexes. Since elongasome disruption by knockdown of *mreB* or *rodA* expression causes localized bulging at the known *B. burgdorferi* cell zones (99), we believe that the elongasome is responsible for cell elongation at these locations, which implies that *B. burgdorferi* bactofilin and elongasome activities are coordinated. Studies in *A. biprosthecum* and *H. neptunium* (79, 87) have established that bactofilins can corral elongasome activity and constrain it to discrete subcellular locations. Ascribing a similar role to *B. burgdorferi* BbbF and BbbG would explain our observation that peptidoglycan insertion becomes delocalized but is not blocked following bactofilin depletion.

Studies of other bacteria have established that elongasomes remain active under experimental conditions that abrogate MreB function and cause rod-to-round cell shape transitions by synthesizing misoriented glycan chains (57, 58, 115–117). Thus, the lack of *B. burgdorferi* cell bulging when bactofilin expression is downregulated indicates either that elongasome activity, while delocalized, remains properly oriented by MreB, or that elongasome-independent peptidoglycan synthases insert the new material. In contrast, cells in which we knocked down *mreB* expression inserted new peptidoglycan within the bulges forming at the established zones of *B. burgdorferi* growth and mCherry-BbbG localized at these bulges (Fig. S12B), which suggests that BbbF and BbbG function upstream of the elongasome. In this scenario, the time needed to assemble and activate elongasomes (37) may also explain the delay between bactofilin accumulation and new peptidoglycan insertion (Fig. 5F and G). Control of the *B. burgdorferi* MreB-driven elongasome by BbbF and BbbG would represent an evolutionarily divergent mechanism from those imposing spatial separation between bactofilin and elongasome activities in *H. pylori* (96) or between bactofilin activity and the MreB-driven, specialized divisome of *C. trachomatis* (85, 118–121).

In addition to spatial control of peptidoglycan insertion, BbbF and BbbG may also play a role in peptidoglycan degradation. A recent study revealed an evolutionarily conserved pairing between bactofilins and M23 endopeptidases homologous to *H. neptunium* LmdC (87). While not universal (85), this pairing was observed in several other bacteria, including several *Borreliaceae* species (79, 87, 89, 90, 95). The conserved pairing of homologous genes across species can suggest a functional relationship between the encoded proteins (122). In *B. burgdorferi*, the M23 endopeptidase is encoded by gene *bb0246*, which is located immediately upstream of *bbbG* (Fig. S2A). It is possible that BbbG-associated BB0246 locally degrades peptidoglycan, which could facilitate new peptidoglycan insertion. In this scenario, the cell envelope instability caused by bactofilin depletion may result from spatial uncoupling of peptidoglycan synthesis and peptidoglycan degradation, or disruptions to periplasmic and outer membrane components that interact with the peptidoglycan sacculus. Weakening of the cell wall could explain the bleb formation we observed. In contrast, spatial restriction of peptidoglycan insertion and degradation by bactofilins would allow for joint control over these processes, thus preserving a regular pattern of growth, cellular integrity, and, by extension, ensuring viability.

Altogether, our study demonstrated that bactofilins BbbF and BbbG carry out essential functions in the Lyme disease spirochete, *B. burgdorferi*. We showed that they support cell growth, morphology, and integrity by regulating the spatial patterning of peptidoglycan growth. Our initial characterization of BbbF and BbbG’s involvement in these processes opens avenues for mechanistic investigations of co-regulation of *B. burgdorferi* growth and cellular replication by bactofilins, the elongasome, and the divisome.

## MATERIALS AND METHODS

### Strains and growth conditions

The *B. burgdorferi* strains used in this study are listed in Table 1. They were grown in complete Barbour-Stoenner-Kelly (BSK)-II medium at 34°C under 5% CO_2_ atmosphere (123–125). The BSK-II medium contained 50 g/L bovine serum albumin (Millipore, cat. no. 810036), 9.7 g/L CMRL-1066 (US Biological, cat. no. 5900-01), 5 g/L neopeptone (Difco, cat. no. 211681), 2 g/L yeastolate (Difco, cat. no. 255772), 6 g/L HEPES (Millipore, cat. no. 391338 or Fisher, cat. no. BP310), 5 g/L glucose (Sigma, cat. no. G7021 or Fisher, cat. no. BP350), 0.7 g/L sodium citrate (Sigma, cat. no. C7254 or Fisher, cat. no. BP327), 0.8 g/L sodium pyruvate (Sigma, cat. no. P5280 or Fisher cat. BP356), 0.4 g/L N-acetylglucosamine (Sigma, cat. no. A3286 or Thermo Scientific, cat. no. A13047.09), 2.2 g/L sodium bicarbonate (Sigma, cat. no. S3817 or S5761 or Fisher, cat. no. BP328), and 60 mL/L heat-inactivated rabbit serum (Gibco, cat. no. 161120). The pH of the medium was adjusted to 7.60 with 5 M sodium hydroxide. Occasionally, the medium was supplemented with 10 g/L gelatin. To avoid loss of endogenous plasmids in stationary phase (29), cultures were maintained in exponential growth, with passages performed at densities below 5 × 10^7^ cells/mL, unless otherwise indicated. Cell density was measured by direct counting of samples placed in disposable hemocytometers and observed on a microscope using darkfield illumination, as described before (126). As needed, isopropyl-β-D-1-thiogalactopyranoside (IPTG, Fisher, cat. no. BP1755 or American Bioanalytical, cat. no. AB00841) was added from a 1 M stock in water to make the indicated final concentrations. Final antibiotic concentrations were as follows: streptomycin, 100 μg/mL (127); gentamicin, 40 μg/mL (128); kanamycin, 200 μg/mL (129); and blasticidin S, 10 μg/mL (126).

**TABLE 1:** Strains used in this study

Strain name	Record no.	Relevant genotype	Growth conditions^*a*^	Source or reference
S9	CNTs005	B31-A3-68 Δ*bbe02*::*P*_*flaB*_*-aadA*	Sm	(130)
B31-68-LS	CNTs017	B31-A3-69 Δ*bbe02*::[*P*_*flgB*_-*lacI^Bb^*_*P*_*flgB*_*-aadA*]	Sm	(109)
VCbb08	CNTs173	B31-68-LS Δ*bbbG*::[*P*_*flaB*_*-aacC1_P*_*flac*_*-bbbG*]	Sm, IPTG	This study
VCbb12	CNTs177	B31-68-LS / pBSV2_P_flac_-BbbG	Km, Sm	This study
VCbb07	CNTs172	B31-68-LS Δ*bbbG*::*P*_*flgB*_*-aacC1* / pBSV2_P_flac_-BbbG	Km, Sm, IPTG	This study
CJW_Bb176	CNTs155	S9 / pBSV2_P_0826_-mCherry^Bb^-BbbG	Km, Sm	This study
CJW_Bb279	CNTs156	VCbb08 / pBSV2_P_0826_-mCherry^Bb^-BbbG_P_0026_-RBS-mEGFP^Bb^	Km, Sm, IPTG	This study
CNTs191	CNTs191	B31-68-LS Δ*bbbF*::[r(*P*_*flaB*_*-aphI*)_*P*_*pQE30*_*-bbbF*]	Sm, IPTG	This study
CNTs198	CNTs198	CNTs191 / pBSV2G_P_0526_-BbbF	Sm, Gm, IPTG	This study
CNTs216	CNTs216	S9 / pBSV2G_P_0526_-mCherry^Bb^-BbbF	Sm, Gm	This study
CNTs217	CNTs217	CNTs191 / pBSV2G_P_0526_-mCherry^Bb^-BbbF	Sm, Gm, IPTG	This study
CNTs220	CNTs220	CNTs191 / pBSV2B_P_0826_-mCherry^Bb^-BbbG	Sm, Bs, IPTG	This study
CJW_Bb181	CNTs148	S9 / pBSV2G_P_0826_-mCherry^Bb^	Sm, Gm	This study
CNTs287	CNTs287	S9 / pBSV2B_P_0826_-mCherry^Bb^-BbbG_1-10_	Sm, Bs	This study
CNTs288	CNTs288	S9 / pBSV2B_P_0826_-mCherry^Bb^-BbbG_1-21_	Sm, Bs	This study
CNTs315	CNTs315	CNTs191 / pBSV2B_P_0826_-msfGFP^Bb^-FtsA	Sm, Bs, IPTG	This study
CNTs336	CNTs336	VCbb07 / pBSV28-1B_P_0826_-msfGFP^Bb^-FtsA	Sm, Bs, IPTG	This study
CNTs318	CNTs318	K2 / pBbdCas9S(RBSmut)_P_syn_-sgRNAmreB_P_0826_-mCherry^Bb^-BbbG	Km, Sm	This study

^*a*^ Sm, streptomycin; Km, kanamycin; Gm, gentamicin; Bs, blasticidin S.

The *Escherichia coli* strains used in this study were propagated at 30°C on Luria-Bertani agar plates or in Super Broth (35 g/L bacto-tryptone, 20 g/L yeast extract, 5 g/L sodium chloride, 6 mM sodium hydroxide) liquid cultures with shaking. Antibiotics were used as follows: gentamicin at 15 to 20 μg/mL, streptomycin or spectinomycin at 50 μg/mL, rifampicin at 50 μg/mL (plate) or 25 μg/mL (liquid culture), and kanamycin at 50 μg/mL. Most often, NEB 5-alpha (New England Biolabs, cat. no. C2987) was used as a host for genetic manipulations and archiving constructs, except that plasmids containing IPTG-inducible promoters were generated and maintained in NEB 5-alpha F*′ Iq* hosts (New England Biolabs, cat. no. C2992) (99). Transformants were obtained by heat shock and then allowed to recover for 1 h with shaking at 30°C in SOC medium (20 g/L tryptone, 5 g/L yeast extract, 10 mM NaCl, 2.5 mM KCl, 10 mM MgSO_4_, and 20 mM glucose) or Super Broth before plating.

### Plasmid construction methods

The plasmids used in this study are listed in Table 2, and the sequences of the oligonucleotide primers used in their construction are provided in Table 3. The construction methods are described below. Standard molecular biology techniques were used unless otherwise indicated. TOPO cloning kits were purchased from Invitrogen. Restriction endonucleases were purchased from New England Biolabs.

**TABLE 2:** Plasmids used in this study

Plasmid	Description	Antibiotic resistance^*a*^	Source or reference
pCR2.1-TOPO	Cloning vector	Am, Km	Invitrogen
pCR-XL-TOPO	Cloning vector	Zn, Km	Invitrogen
pCR-BluntII-TOPO	Cloning vector	Zn, Km	Invitrogen
pBSV2	*E. coli* – *B. burgdorferi* shuttle vector	Km	(131)
pBSV2G	*E. coli* – *B. burgdorferi* shuttle vector	Gm	(128)
pBSV28-1	Shuttle vector with plasmid maintenance locus from linear plasmid 28-1	Km	(132)
pTAflacp	Cloning vector containing *P*_*flac*_ (*flacP*) promoter	Am, Km	(107)
pBSV2G_2	*E. coli* – *B. burgdorferi* shuttle vector	Gm	(126)
pBSV2_2	*E. coli* – *B. burgdorferi* shuttle vector	Km	(126)
pBSV2B	*E. coli* – *B. burgdorferi* shuttle vector	Bs, Rf	(126)
pKIKan_idCas9_Chr_center	Plasmid containing the promoter *P*_*pQE30*_	Km	(99)
pBbdCas9S(RBSmut)_P_syn_-sgRNAmreB	All-in-one inducible CRISPRi shuttle vector targeting *mreB*	Sm	(99)
pKI_Kan	Plasmid containing kanamycin resistance gene *aphI*	Km	(114)
pdel_parS	Suicide vector plasmid	Gm	(114)
pBSV2G_P_0826_-mCherry^Bb^-ParB	Plasmid containing *P*_*0826*_*-mcherry*^*Bb*^ fusion.	Gm	(114)
pBLJ084	Shuttle vector expressing *msfgfp^Bb^-ftsA*	Km	(100)
pΔbbbG::P_flgB_-AacC1	Suicide vector for deletion of *bbbG*	Gm	This study
piBbbG	Suicide vector for replacing native *bbbG* gene with an inducible version	Gm	This study
pBSV2_P_flac_-BbbG	Shuttle vector for inducible expression of *bbbG*	Km	This study
pBSV2_P_0026_-mEGFP^Bb^	Shuttle vector containing an *megfp^Bb^* expression cassette.	Km	This study
pBSV2_P_0826_-mCherry^Bb^-BbbG	Shuttle vector expressing *mcherry^Bb^-bbbG*	Km	This study
pBSV2_P_0826_-mCherry^Bb^-BbbF	Shuttle vector expressing *mcherry^Bb^-bbbF*	Km	This study
pBSV2_P_0826_-mCherry^Bb^-BbbG_P_0026_-RBS-mEGFP^Bb^	Shuttle vector expressing *mcherry^Bb^-bbbG* and free *megfp^Bb^*.	Km	This study
pKI_r(P_flaB_-AphI)_P_pQE30_-BbbF	Suicide vector for replacing native *bbbF* gene with an inducible version	Km	This study
pBSV2G_P_0526_-BbbF	Shuttle vector expressing untagged *bbbF*	Gm	This study
pBSV2G_P_0526_-mCherry^Bb^-BbbF	Shuttle vector expressing *mcherry^Bb^-bbbF*	Gm	This study
pBSV2B_P_0826_-mCherry^Bb^-BbbG	Shuttle vector expressing *mcherry^Bb^-bbbG*	Bs, Rf	This study
pBSV2B_P_0826_-mCherry^Bb^-BbbG_1-10_	Shuttle vector expressing *mcherry^Bb^* fused to *bbbG* N-terminal fragment	Bs, Rf	This study
pBSV2B_P_0826_-mCherry^Bb^-BbbG_1-21_	Shuttle vector expressing *mcherry^Bb^* fused to *bbbG* N-terminal fragment	Bs, Rf	This Study
pBSV2B_P_0826_-msfGFP^Bb^-FtsA	Shuttle vector expressing *msfgfp^Bb^-ftsA*	Bs, Rf	This Study
pBSV28-1B_P_0826_-msfGFP^Bb^-FtsA	Shuttle vector expressing *msfgfpP^Bb^-ftsA*	Bs, Rf	This Study
pBbdCas9S(RBSmut)_P_syn_-sgRNAmreB_P_0826_-mCherry^Bb^-BbbG	All-in-one inducible CRISPRi shuttle vector targeting *mreB*, with a constitutively expressed *mcherry^Bb^-bbbG*	Sm	This Study

^*a*^ Km, kanamycin; Gm, gentamicin; Am, ampicillin; Zn, zeocin; Bs, blasticidin S; Rf, rifampicin.

**TABLE 3:** Oligonucleotide primers used in this study

Name	Sequence (5′−3′)
VCp1	GCTAGCGATCTTAAAAATATTAAAAGC
VCp2	CTCGAGGTCTTACACCCAATTCACATTAGC
VCp3	GTCGACAGACGGGGCTTTTCTTTC
VCp4	CCGCGGCAATAAGGTGCATCAAGAAG
VCp5	GTCGACCTAATACCCGAGCTTCAAGGAG
VCp6	CCGCGGGAACGAATTGTTAGGTGGC
VCp7	ACGCGTAAAAGCAAAATAGAAAGTTC
VCp8	GTCGACCTATTTTGAAGCTCCTGT
VCp9	AACAGGAGCTTCAAAATAGGTCGACGTCGACTGTCTGTCGCCTC
VCp10	TATGAACGTTAACGTTTTAGGTGGCGGTAC
VCp11	CATATGTTAAATTTTTTGACTGTAAAAAAAG
VCp12	ACTAGTTTATTTACTTTCATCTATTTTGTTCAC
VCp13	TCTAGATGCATGCTCGAGCGGCC
VCp14	AAGCTTGGTACCGAGCTCGGATCCAC
par.5	AGAGTCGACATGTTAAATTTTTTGACTGTAAAAAAAGAAAGAAAAGCCC
par.6	TGCCTGCAGTTATTTACTTTCATCTATTTTGTTCACAACACTTG
par.9	AGAGTCGACATGAATGCTTTAACTTTAAAGCATTCATTTTTAGAGG
par.10	TGCCTGCAGTTATTCTTCTAAATCCTCCATTTCACAATTCCC
NT160	CGCAAGCTTATTTATATAATTCATCCATACCATGAGTAATACC
NT341	TATGGATCCAGGAGGTTCATGAGTAAAGGTGAAGAATTATTTACTGG
CNTp0171	GAAATCCAATAACCATTCTTCTCTGAATGCATCTAGAGGGCCCAATTCGCCC
CNTp0172	ATATTATCAGCAGATTTTTTATCCTGATCCGAGCTCGGTACCAAGCTTGATGC
CNTp0177	GCAAATAAATTTTTTATGATTTTCTGGGTGTCTGTCGCCTCTTGTGGCTTCC
CNTp0178	AAAAAATCATAAAAAATTCAATCAAAAGGGTAAAAAAACAAAAGATCCTTTAAAGG
CNTp0180	GGAAGCCACAAGAGGCGACAGACACCCAGAAAATCATAAAAAATTTATTTGC
CNTp0181	ATCAAGCTTGGTACCGAGCTCGGATCAGGATAAAAAATCTGCTGATAATATGG
CNTp0182	ATCTTTTGTTTTTTTACCCTTTTGATTGAATTTTTTATGATTTTTTTATTATTTTAATCG
CNTp0187	GAGAAATTACATATGAGTATAGATAGCTTAGAATTCG
CNTp0188	CTAGATGCATTCAGAGAAGAATGGTTATTGG
CNTp0189	GAATTCTAAGCTATCTATACTCATATGTAATTTCTCCTCTTTAATGAATTCTGTGTG
CNTp0230	TTCGGATCCAGGAGGTTCATGAGTATAGATAGCTTAGAATTCGAAGAA
CNTp0231	TTCCCTAGGTCATTCTTCTAAATCCTCCATTTCAC
CNTp0544	CCAACTTTTTTACAAAAAATTTTTTATTAAAACACTTAGG
CNTp0545	CCTAAGTGTTTTAATAAAAAATTTTTTGTAAAAAAGTTGG
CNTp0546	TAAAAAAAGAATAACTGCAGGCATGCAAGCTTGG
CNTp0547	TGCATGCCTGCAGTTATTCTTTTTTTACAGTCAAAAAATTTAACATGTCG
CNTp0548	TTTTTGATGAATAACTGCAGGCATGCAAGCTTGG
CNTp0549	TGCCTGCAGTTATTCATCAAAAATAAACAAAGACGGG
CNTp0864	GGGTACCGATGGCAATAGAAGAATCTATAGAAAGC
CNTp0865	ACTCTAGAGGTCAAAACCATTCTTTCAAAAACC
CNTp0866	ATGGTTTTGACCTCTAGAGTCGACCTGC
CNTp0867	TTCTATTGCCATCGGTACCCCGGGATCC
CNTp0870	TTCTATTGCCGATCAGATCTCAGCTTTTTTTTG
CNTp0871	AAGTAAATAAAAGCTTTTATTAGTCACCTCCTAG
CNTp0872	AATAAAAGCTTTTATTTACTTTCATCTATTTTGTTCACAAC
CNTp0873	AGATCTGATCGGCAATAGAAGAATCTATAGAAAGC
CNTp0975	GCTTACGCGTCACGTGCTAAAACTTCATTTTTAATTTAAAAG
CNTp0976	GTACCCCGGGGGTACCGAGCTCGAATTC
CNTp0977	GCTCGGTACCCCCGGGGTACCGATGGCAATAG
CNTp0978	TTAGCACGTGACGCGTAAGCCGATCTCGG

#### pΔbbbG::P_flgB_-aacC1

A fragment of the *B. burgdorferi* chromosome was PCR-amplified with primers VCp1 and VCp2 and cloned into plasmid pCR2.1-TOPO. In the resulting intermediate plasmid, inverse PCR was performed with primers VCp3 and VCp4 to delete the central portion of gene *bbbG* (*bb0245*). The product was subcloned into pCR-XL-TOPO. The resulting plasmid was then digested with SalI and SacII to release the pCR2.1-TOPOΔbbbG backbone. The P*_flgB_-aacC1* gentamicin resistance cassette was PCR-amplified from pBSV2G using VCp5 and VCp6, subcloned into pCR-BluntII-TOPO, then released as a SalI/SacII fragment and ligated into the SalI/SacII-digested pCR2.1-TOPOΔbbbG backbone described above. The product was digested with AclI then re-ligated to inactivate the ampicillin resistance gene, yielding plasmid pΔbbbG::P_flgB_-AacC1.

#### piBbbG

A fragment of gene *bb0246* was PCR-amplified using primers VCp7 and VCp8 and cloned into pCR-BluntII-TOPO. A P*_flaB_-aacC1* gentamicin resistance cassette previously described (109) was PCR-amplified using primers VCp9 and VCp10 and cloned into pCR-BluntII-TOPO. The two plasmids were digested with SalI and SpeI, and the P*_flaB_-aacC1* fragment was ligated to the pCR-BluntII-TOPO_bb0246 backbone. The *bbbG* sequence was PCR-amplified using primers VCp11 and VCp12 and cloned into pCR-BluntII-TOPO; then, *bbbG* was released as an NdeI/SpeI fragment and cloned into the NdeI/SpeI-digested pTAflacp (107). The resulting P*_flac_-bbbG* fragment was released as an XbaI/KpnI fragment and ligated to the SpeI/KpnI backbone of pCR-BluntII-TOPO_bb0246_P_flaB_-aacC1 to yield plasmid piBbbG.

#### pBSV2_P_flac_-BbbG

A P*_flac_-bbbG* fragment described above was PCR-amplified using VCp13 and VCp14 and cloned in pCR-BluntII-TOPO; then, released as a BamHI/XbaI fragment and finally cloned into BamHI/XbaI-digested pBSV2.

#### pBSV2_P_0826_-mCherry^Bb^-BbbG and pBSV2_P_0826_-mCherry^Bb^-BbbF

Through intermediate constructs, the following fragments were assembled in the pBSV2_2 backbone (126). A P*_0826_-mcherry^Bb^* transcriptional fusion lacking a STOP codon was previously described in plasmid pBSV2G_P_0826_-mCherry^Bb^-ParB (114) and was inserted between the SacI and KpnI sites of pBSV2_2. The bactofilin genes were amplified using primers par.5 and par.6 (*bb0245*/*bbbG*) or par.9 and par.10 (*bb0538*/*bbbF*). The resulting fragments were digested with SalI and PstI and inserted into the corresponding sites of pBSV2_2. The mCherry-bactofilin fusions thus generated contain a GYRSSRVD linker, with the mCherry-BbbF linker including the 14 amino acids preceding the likely BbbF translational start site (Fig. S3D).

#### pBSV2_P_0826_-mCherry^Bb^-BbbG_P_0026_-RBS-mEGFP^Bb^

The following steps were taken to generate pBSV2_P_0026_-RBS-mEGFP^Bb^: promoter P*_0026_* (126), flanked by SacI and BamHI sites, and *megfp^Bb^* (126), PCR-amplified using primers NT160 and NT341 and digested with BamHI and HindIII, were assembled between the SacI and HindIII sites of plasmid pBSV2_2. A fragment containing the P*_0026_-RBS-megfp^Bb^* sequence was released from pBSV2_P_0026_-RBS-mEGFP^Bb^ as a BsrBI/AvrII fragment and then ligated into the FspI/AvrII backbone of pBSV2_P_0826_-mCherry^Bb^-BbbG.

#### pKI_r(P_flaB_-aphI)_P_pQE30_-BbbF

A region of the *B. burgdorferi* chromosome upstream of *bbbF*, encompassing nucleotides 550,492 to 548,886, was PCR-amplified with primers CNTp0181 and CNTp0182. A region of the *B. burgdorferi* chromosome encompassing nucleotides 548,843 to 547,208 and containing *bbbF* (beginning at the second annotated start site, Fig. S3D) and downstream genes were PCR-amplified with primers CNTp0187 and CNTp0188. Both amplification reactions were done using genomic DNA from strain CJW_Bb378 (114). The antibiotic resistance cassette P*_flaB_-aphI* was amplified from pKI_Kan (114) using primers CNTp0178 and CNTp0177. The synthetic promoter P*_pQE30_* was amplified from plasmid pKIKan_idCas9_Chr_center (99) using primers CNTp0180 and CNTp0189. The backbone was amplified using primers CNTp0171 and CNTp0172 from a derivative of pdel_parS (114). Fragments were assembled using the NEBuilder HiFi assembly kit.

#### pBSV2G_P_0526_-BbbF

The gene *bbbF* was amplified from B31-68-LS using primers CNTp0230 and CNTp0231, and the resulting PCR product was digested with BamHI and AvrII. Promoter P*_0526_* was previously described (126) and is flanked by SacI and BamHI sites. Through intermediary constructs, the SacI/BamHI P*_0526_* fragment and the BamHI/AvrII *bbbF* fragment were assembled between the SacI/AvrII sites of pBSV2G_2 (126).

#### pBSV2G_P_0526_-mCherry^Bb^-BbbF

*mcherry^Bb^-bbbF* was released from pBSV2_P_0826_-mCherry^Bb^-BbbF with BamHI and AvrII and ligated into the BamHI/AvrII-linearized backbone of pBSV2G_P_0526_-BbbF.

#### pBSV2B_P_0826_-mCherry^Bb^-BbbG

P*_0826_-mcherry^Bb^-bbbG* was released from plasmid pBSV2_P_0826_-mCherry^Bb^-BbbG with BsrBI and AvrII and ligated into the BsrBI/AvrII-linearized backbone of pBSV2B (126).

#### pBSV2B_P_0826_-mCherry^Bb^-BbbG_1-10_

A fragment of pBSV2B_P_0826_-mCherry^Bb^-BbbG, including the first 30 nucleotides of *bbbG*, was PCR-amplified with primers CNTp0544 and CNTp0547. A second fragment of pBSV2B_P_0826_-mCherry^Bb^-BbbG containing the stop codon of *bbbG* was PCR-amplified using primers CNTp0545 and CNTp0546. These fragments were assembled using the NEBuilder HiFi assembly kit.

#### pBSV2B_P_0826_-mCherry^Bb^-BbbG_1-21_

A fragment of pBSV2B_P_0826_-mCherry^Bb^-BbbG, including the first 63 nucleotides of *bbbG*, was PCR-amplified with primers CNTp0544 and CNTp0549. A second fragment of pBSV2B_P_0826_-mCherry^Bb^-BbbG containing the stop codon of *bbbG* was PCR-amplified using primers CNTp0545 and CNTp0548. These fragments were assembled using the NEBuilder HiFi assembly kit.

#### pBSV2B_P_0826_-msfGFP^Bb^-FtsA

The insert P*_0826_-msfgfp^Bb^-ftsA* was amplified from pBLJ084 (100) using primers CNTp0864 and CNTp0865. We specify the use of monomeric superfolder GFP (msfGFP^Bb^) in our naming to distinguish from other GFP variants used elsewhere. The pBSV2B backbone was PCR-amplified using primers CNTp0866 and CNTp0867. These fragments were assembled using the NEBuilder HiFi assembly kit.

#### pBSV28-1B_P_0826_-msfGFP^Bb^-FtsA

The insert P*_0826_-msfgfp^Bb^-ftsA* and blasticidin resistance were PCR-amplified from pBSV2B_P_0826_-msfGFP^Bb^-FtsA using primers CNTp0977 and CNTp0978. The backbone of pBSV28-1 (132) was PCR-amplified using primers CNTp0975 and CNTp0976. These fragments were assembled using the NEBuilder HiFi assembly kit.

#### pBbdCas9S(RBSmut)_P_syn_-sgRNAmreB_P_0826_-mCherry^Bb^-BbbG

The plasmid pBbdCas9S(RBSmut)_P_syn_-sgRNAmreB (99) was linearized and amplified using primers CNTp0870 and CNTp0871. The insert *P_0826_-mCherry^Bb^-bbbG* was amplified using primers CNTp0872 and CNTp0873 from pBSV2B_P_0826_-mCherry^Bb^-BbbG. These fragments were assembled using the NEBuilder HiFi assembly kit.

### *B. burgdorferi* strain generation

*B. burgdorferi* competent cells were prepared as described before (133). Cultures were allowed to grow to densities between 2 × 10^7^ cells/mL and 10^8^ cells/mL. The cells were centrifuged for 10 min at 10,000 × *g* and 4°C and resuspended in ice-cold electroporation solution (EPS), which contained 93.1 g/L sucrose (American Bioanalytical, cat. no. AB01900) and 150 mL/L glycerol (American Bioanalytical, cat. no. AB00751). A total of three centrifugation steps followed by resuspension in decreasing volumes of EPS achieved the desired salt removal and concentration of the cells. Aliquots of 50 to 100 μL of concentrated *B. burgdorferi* cells in EPS were mixed with DNA and immediately electroporated in a 2 mm gap cuvette (Bio-Rad) using the following settings: 2.5 kV, 25 μF, and 200 Ω (134, 135). Shuttle vector DNA was used at 15–50 μg per transformation. Suicide vector DNA (50–75 μg per transformation) was linearized by restriction endonuclease digestion and then ethanol precipitated (136). Electroporated *B. burgdorferi* cells were resuspended in BSK-II medium, supplemented with 100 μM IPTG as needed, and allowed to recover until the next day. Transformants were selected by semisolid BSK-agarose plating under antibiotic selection, as previously described (99). Single colonies were removed from the plates and expanded in BSK-II medium. Retention of the full parental plasmid complement was confirmed for each clone by multiplex PCR (137). However, strains transformed with pBSV28-1B_P_0826_-msfGFP^Bb^-FtsA lost lp28-1, which was displaced by the incompatible pBSV28-1-derived shuttle vector (132). Suicide vector transformants were checked for correct recombination by PCR. To confirm successful shuttle vector transformations, 14 mL cultures were pelleted at 10,000 × *g* for 10 min, and the pellet was then resuspended in 500 µL water and miniprepped using the Zyppy plasmid miniprep kit (Zymo, cat. no. D4020). The resulting DNA was used to transform NEB 5-alpha or NEB 5-alpha F′ lq *E. coli* competent cells (New England Biolabs). Colonies were selected using appropriate antibiotics, grown, miniprepped, and whole-plasmid sequenced using Nanopore technology at Quintara Biosciences.

### RNA isolation and qRT-PCR

*B. burgdorferi* cultures of strains VCbb07, VCbb08, and CNTs191 were maintained in exponential growth in the presence of 100 μM IPTG. The cells were pelleted by centrifugation for 10 min at 4,300 × *g*, resuspended in 1 mL IPTG-free BSK-II medium, counted to determine cell density, and diluted to 10^6^ cells/mL in 30 mL cultures with or without 100 μM IPTG. Cells were harvested 24 h later by centrifugation (10 min at 4,300 × *g*). The pellet was resuspended in 400 μL buffer HN (138) and added to 1 mL RNAProtect Bacteria (Qiagen, cat. no. 76506), vortexed, incubated for 5 min at room temperature, and then centrifuged for 10 min at 10,000 × *g*. The liquid was removed by aspiration, and the pellet was stored at −80°C. The RNA was released from the pellet using enzymatic lysis and proteinase K digestion performed as recommended by protocol 4 in the RNAprotect bacteria reagent manual. This was followed by purification using the RNeasy Mini Kit (Qiagen, cat. no. 74106). Trace DNA was removed using the TURBO DNA-free kit (Thermo Fisher Scientific, cat. no. AM1907). The concentration of the RNA was determined using NanoDrop OneC, and the RNA was stored at −80°C.

Quantitative reverse transcriptase PCR (qRT-PCR) was performed using the Verso 1-Step RT-qPCR Kit (Thermo Scientific, cat. no. AB4105C). The RNA was diluted to 10 ng/μL in water, and 1 μL was used for each reaction. Two technical duplicate reactions were performed for each sample. An additional no RT control lacked the RT enzyme provided with the kit. The reaction volume was 25 μL/well. Cycling was done on a Bio-Rad qRT-PCR system (CFX Duet) under the following conditions: reverse transcription, 15 min at 50°C; denaturation, 15 min at 95°C; 40 cycles each consisting of 15 s at 95°C, 30 s at 50°C, and 30 s at 72°C with fluorescence reading in the SYBR channel; and a melting curve from 65°C to 95°C in 0.5°C increments. The primers used for qRT-PCR are listed in Table 4. Quantification cycle (*C*_*q*_) values and direct observation of amplification curves obtained in the absence of reverse transcription confirmed that the measured RNA amounts were not due to DNA contamination. Relative transcript amounts were determined by the ΔΔ*C*_*q*_ method (139) and were normalized to the levels of the *flaB* transcript present in each sample.

**TABLE 4:** Primers used for qRT-PCR

Gene	Forward primer	Reverse primer	Reference
*flaB*	TTCAATCAGGTAACGGCACA	GACGCTTGAGACCCTGAAAG	(140)
*bb0246*	TCCTTCTCTTTGGCCTCTTG	CCCCAAGATCTATGCCTTTG	This study
*bbbG*	TTGCATCAGGGTGTGTTATTG	GCGCCAATATCCATATACCC	This study
*bb0244*	CTTTTGAGTGAGCCTTCCAGAC	TTCTATACTTGGGGCGCTTG	This study
*bb0539*	CTGCAACAGCTCTTTTCTTGC	CCAGCAGTAATTCCGAATGTG	This study
*bbbF*	GGAAAAAGCTGATGTTGAAGC	CCTATTAATTTGCCGGTTTTG	This study
*bb0537*	AGCCGATTTTGCATACGAAC	TTTGCATTTATTCTTGATCTTGC	This study

### RNA sequencing

RNA sequencing (RNA-seq) on *B. burgdorferi* strain B31-68-LS was performed previously (110). Briefly, total RNA was extracted from *B. burgdorferi* cells grown to culture densities between 5 × 10^7^ and 7 × 10^7^ cells/mL using TRIzol reagent (Thermo Fisher Scientific, cat. no. 15596026) and treated with 1 unit of Ambion DNAse I (Thermo Fisher Scientific, cat. no. AM2222) for 10 min at 37℃. RNA was quantified and assessed for quality, and RNA samples possessing RNA integrity number (RIN) values above 7.4 were used for sequencing. Ribosomal RNA was depleted using the QIAseq FastSelect −5S/16S/23S kit (Qiagen, cat. no. 335925) before next-generation sequencing library preparation with the TruSeq Stranded mRNA-Seq Sample Preparation Kit (Illumina, cat. no. 20020595). Libraries were sequenced on an Illumina NextSeq 550 at 2 × 75 bp paired-end using a Mid Output 150 cycle kit (Illumina, cat. no. 20024904). RNA-seq reads were compiled, filtered, and aligned to the *B. burgdorferi* B31 genome (RefSeq AE000783-AE000794 and AE001575-AE001584) using bowtie2 (141). Reads for annotated genes were quantified using featureCounts (142, 143). The RPKM (reads per kilobase per million reads) were then calculated to assess gene expression.

### Isolation of escape mutants

*B. burgdorferi* cultures of strains VCbb07 and VCbb08 were maintained in exponential growth in the presence of 100 μM IPTG. The cells were pelleted by centrifugation for 10 min at 4,300 × *g*, resuspended in 1 mL IPTG-free BSK-II medium, counted to determine cell density, and diluted to 10^5^ cells/mL in 6 mL cultures without IPTG. To obtain clonal isolates, cultures were left to grow to ~10^7^ cells/mL in liquid media, then plated on semi-solid BSK agarose without IPTG. Following colony growth, clonal isolates were picked and grown in 14 mL liquid BSK-II medium to a density of ~10^7^ cells/mL with or without IPTG. Cells were pelleted by centrifugation for 10 min at 4,300 × *g*, then resuspended in 180 µL Buffer ATL (Qiagen, cat. no. 939016) followed by the “Purification of Total DNA from Animal Tissues (SpinColumn Protocol)” using the DNEasy Blood and Tissue Kit (Qiagen, cat. no. 69504). The DNA was eluted in water and stored at −20°C.

### Whole-genome sequencing

Whole-genome sequencing of VCbb07 and VCbb08 parental strains and escape mutants was performed by Plasmidsaurus using their Oxford Nanopore sequencing option. To serve as an assembly reference, the *B. burgdorferi* B31 genome (GCF_000008685.2) was downloaded from NCBI and manually modified to include the modifications made to plasmid lp25, the chromosome, and/or a shuttle vector as needed. Samples were mapped and consensus sequences generated with SAMtools version 1.22.1 (144). Visualization of sequences was done with IGV v2.19.4 (145) or Geneious 2020.0.4. The code (nanopore_analysis_script.sh) used to map reads to the reference genomes was obtained using generative AI (Claude AI version 4.0).

### Scanning electron microscopy

Strain VCbb08 was grown in culture medium supplemented with 100 µM IPTG or without IPTG supplementation for 48 h. Cells were fixed in 2% paraformaldehyde and 2.5% glutaraldehyde in 0.1 M Sorenson’s phosphate buffer for 30 min. Cells were then adhered to poly-L-lysine–coated silicon chips (Electron Microscopy Sciences). Chips were processed as previously described (91): they were fixed in 0.5% OsO_4_, 0.8% K_4_Fe(CN)_6_ in 0.1 M sodium cacodylate buffer (Ted Pella, cat. no. 18851) for 1 h, rinsed in buffer, stained with 1% aqueous tannic acid for 1 h, rinsed with buffer, and then a second fixation with 0.5% OsO_4_ + 0.8% K_4_Fe(CN)_6_ in the same buffer. Chips were then rinsed with distilled water and put through a graduated ethanol series into 100% ethanol. Chips were critically point dried using a Bal-Tec Critical Point Dryer 030 (Leica), mounted on stubs, and sputter coated with 10 nm of iridium (300T, Electron Microscopy Sciences). Images were acquired using a Hitachi SU8000 scanning electron microscope operating at 5 kV.

### Light microscopy

To collect phase-contrast and fluorescence micrographs, *B. burgdorferi* cultures were spotted on a 2% agarose-PBS pad (97, 146) and then covered with a No. 1.5 coverslip. The cells were imaged using Nikon Ti inverted microscopes equipped with 100× Plan Apo 1.45 NA phase-contrast oil objectives, Hamamatsu ORCA-Flash4.0 V2 CMOS cameras, and a Spectra X Light engine (Lumencor) or a Sola LE light source. The microscopes were equipped with the following Chroma filter cubes: DAPI (excitation ET395/25x, dichroic T425lpxr, emission ET460/50m); GFP (excitation ET470/40x, dichroic T495lpxr, emission ET525/50m); and mCherry/TexasRed (excitation ET560/40x, dichroic T585lpxr, emission ET630/75m). Alternatively, images were acquired on a Nikon Ti2-E inverted microscope equipped with a 100× Plan Apo 1.45 NA phase-contrast oil objective, a Hamamatsu Orca-Fusion BT camera, and a Spectra III LED fluorescent light source. The microscope was equipped with a custom filter cube containing a Semrock triple-edge polychroic (Semrock, cat. no. FF409/493/596-Di02-t2−32 x 44-Ti2). Fluorescence images were captured with the excitation and emission filters: DAPI (excitation: 390/22, emission: 432/36), GFP (excitation: 475/28, emission: 525/50), and mCherry/TexasRed (excitation: 575/25, emission: 641/75). Images were acquired using NIS Elements software.

### Image analysis

Light microscopy images were analyzed as follows. First, cell outlines were generated based on the phase-contrast images using the Oufti analysis package (147). Segmentation parameters were adjusted for each image set (Table S1) to optimize the automatically generated outlines. Outlines were curated, which involved: manual removal of outlines of cellular debris, of cells that curled on themselves or crossed other cells, or of cells that were not fully within the image; manual extension of partial cell outlines or manual addition of full cell outlines, when feasible; and manual splitting of cell outlines at sites of clear separation of cytoplasmic cylinders that remained linked by an outer membrane bridge (97). All outlines were refined using the “Refine All” function of the software, followed by a final removal of any improperly rendered outlines. Fluorescence background was subtracted before demographs, and intensity profiles were generated, all using Oufti functions. Total cell fluorescence was calculated based on the final background subtracted and curated cell meshes using MATLAB 2024a. The cell lists were then processed using the MATLAB script addMeshtoCellList.m and CalculateFluorPerCell.m, as previously described (126).

### Data visualization

The following software was used to generate, visualize, and present the data: Nikon NIS Elements software, FiJI (148), GraphPad Prism 10.0.0, Geneious Prime 2020.0.4, Adobe Illustrator 2025, MATLAB 2024a, and Rstudio 4.3.1 with R version 4.3.0 (149) using ggplot2 (150).

### Labeling peptidoglycan insertion

Peptidoglycan insertion was labeled using the fluorescent D-amino acid HADA (Tocris Bioscience cat. 6647), following the procedure previously established in *B. burgdorferi* (97). *B. burgdorferi* cultures were grown under standard conditions described above. Cells were pelleted by centrifugation for 10 min at 4,300 × *g*, resuspended in 1 mL IPTG-free BSK-II medium, counted to determine cell density, and diluted to 10^6^ cells/mL in 6 mL cultures with or without 100 μM IPTG. After 24 h of growth, 990 µL of each culture was mixed with 10 µL 0.1 mM HADA in dimethyl sulfoxide (DMSO) to yield a final concentration of 1 µM HADA and incubated for 1 h at 34°C. Following incubation, the cells were pelleted by centrifugation 8,000 × *g* for 5 min at 4°C and resuspended by pipetting in 1 mL ice-cold BSK-II with no added HADA or IPTG. Two additional washes were performed, with the final pellet resuspended in 30–100 µL ice-cold BSK-II. Cells were imaged as described above.

### Statistical analysis

Cell mesh files containing cell size, shape, and fluorescence data were generated via Oufti as described above. Spot detection (147) was used to find fluorescent signal foci along the cell length. A prominence value of 0.4 over the whole cell mean signal was set for mCherry-BbbF, mCherry-BbbG, and HADA, and a prominence of 0.2 for msfGFP-FtsA. The MATLAB scripts extractFluroescenceDataFlexible.m and detectPositionalPeaks.m, created with generative AI (Claude version 4.5), were used to identify peaks that occurred within set distances from the mid- or quarter-cell positions of a cell. For mCherry-BbbF, mCherry-BbbG, and HADA signals, a window of ± 7.5% of the cell length was used about each position. For msfGFP-FtsA, a window of ± 10% of the cell length was used to account for its more variable pattern (100). Cell length and cell signal category analyses were done using RStudio 4.3.1 with R version 4.3.0 (149). Wilcoxon rank sum tests were done using wilcox.test in the base stats package, and Jonckheere-Terpstra tests were done using Jonckheere.test in package clinfun (151).

### Phylogenetic analyses

Putative spirochete bactofilin protein sequences were identified using NCBI’s Basic Local Alignment Search Tool (BLAST) (152). The search was performed in August 2025. *Leptospira biflexa* bactofilin protein sequences LbbA through LbbE (NCBI RefSeq ID WP_012388301.1, WP_012390385.1, WP_012389582.1, WP_012388418.1, and WP_012388052.1) were used as bait, and all hits within the spirochete phylum were retrieved. Additional searches were performed using *B. burgdorferi* strain B31 bactofilin hits (RefSeq ID WP_002665254.1, WP_002656070.1, and WP_002556844.1). The resulting spirochete bactofilin list was curated to remove duplicate entries. Only RefSeq entries (ID starting with WP) were retained. Additionally, partial sequences were removed. A total of 879 sequences were then assembled into a phylogenetic tree rooted on a Lindowbacteria sequence (MBI4831304), as previously done for other spirochete phylogenetic analyses (153). The tree was generated using the Geneious Prime Tree Builder function with the following settings: alignment type, global alignment with free end gaps; cost matrix, Blosum62; genetic distance model, Jukes-Cantor; tree build method, neighbor-joining, with no outgroup. Sequence alignments were also performed using Geneious Prime Alignment function and the same alignment type and cost matrix.

### Structural predictions

Structural predictions were performed using the AlphaFold 3 platform (105, 154) hosted on Google’s Colaboratory platform (155). The structures predicted by AlphaFold 3 were visualized using UCSF ChimeraX 1.8 (156).

### Western blotting

Cultures of *B. burgdorferi* were grown as described in 45 mL liquid cultures in BSK-II. Conditions requiring 24 h of IPTG depletion were created as described. Culture density was determined by direct counting, and volumes were adjusted for a final input of 3.15 × 10^8^ cells. Cells were washed twice by pelleting (10,000 × *g* for 10 min at 4°C) and resuspension in 1 mL buffer HN containing 5× cOmplete protease inhibitor (Sigma Aldrich cat. 11836153001). Cell pellets were resuspended in 1× LDS sample buffer (Thermo Scientific cat. B0007) containing 5× cOmplete protease inhibitor, 1 mM DL-1,4-dithiothreitol (Thermo Scientific cat. 426380100), and 2.5% (vol/vol) 2-mercaptoethanol, then boiled at 95°C for 5 min prior to SDS-PAGE. Proteins were run on a 4–12% Bis-Tris Plus gel (Thermo Scientific cat. NW04122BOX) in 1× MES-SDS buffer (Thermo Scientific cat. NP0002) at 180 V for 25–30 min. Proteins were transferred to nitrocellulose membranes using an Invitrogen iBlot 2 gel transfer device (transfer settings: 20 V, 7 min). Membranes were washed with 0.1% (vol/vol) Tween-20 in 1× phosphate-buffered saline (PBS-T) and blocked with 4% (wt/vol) bovine serum albumin in PBS-T. For mCherry detection, incubation with a primary anti-mCherry chicken polyclonal antibody (Novus Biologic cat. NBP2-25152) diluted 1:2,000 in blocking buffer was performed overnight at 4°C. Membranes were washed six times for 5 min each with PBS-T, then incubated with a rabbit anti-chicken horseradish peroxidase (HRP)-conjugated secondary antibody (Invitrogen cat. A16130) diluted 1:2,000 in blocking buffer for 1 h at room temperature. For FlaB, both primary and secondary incubations were performed for 1 h at room temperature with a rat anti-FlaB primary (Kerafast cat. ECN013) diluted 1:6,000 in blocking buffer, and a rabbit anti-rat HRP-conjugated secondary (Sigma Aldrich cat. A5795) diluted 1:80,000. Chemiluminescence was detected after treatment with SuperSignal West PICO (Fisher Scientific cat. PI34577) or SuperSignal West FEMTO (Fisher Scientific cat. PI34094) PLUS chemiluminescent substrates using a Bio-Rad Chemidoc gel imager.

## Data Availability

Custom MATLAB scripts for fluorescence analysis (extractFluorescenceDataFlexible.m, detectPositionalPeaks.m), the whole-genome assembly script (nanopore_analysis_script.sh), and R statistical analysis code (bactofilin_peakanalyses.rmd) are deposited at GitHub (https://github.com/TakacsLab/Bactofilin_project.git). Raw nanopore sequencing reads are deposited at NCBI SRA under accession number PRJNA1440507. Fluorescence data used for statistical analyses are provided as supplemental material (Table S6 to S9). Fluorescence microscopy images and associated segmentation files are available from the corresponding author upon reasonable request.

## SUPPLEMENTAL MATERIAL

Supplemental figures jb.00198-26-s0001.pdf Figures S1 to S12.

Supplemental legends jb.00198-26-s0002.docx Legends for Figures S1 to S12 and Tables S1 to S9.

Supplemental tables jb.00198-26-s0003.xlsx Tables S1 to S9.

ASM does not own the copyrights to Supplemental Material that may be linked to, or accessed through, an article. The authors have granted ASM a non-exclusive, world-wide license to publish the Supplemental Material files. Please contact the corresponding author directly for reuse.
