# Supplementary material for: Bactofilins are essential spatial organizers of peptidoglycan insertion in the Lyme disease spirochete Borrelia burgdorferi
Source: J Bacteriol. 2026 Jun 29;208(7):e00198-26. doi: 10.1128/jb.00198-26 (PMC13393453; doi:10.1128/jb.00198-26)
Supplement: Supplemental figures — Figures S1 to S12. [file jb.00198-26-s0001.pdf]

Figure S1

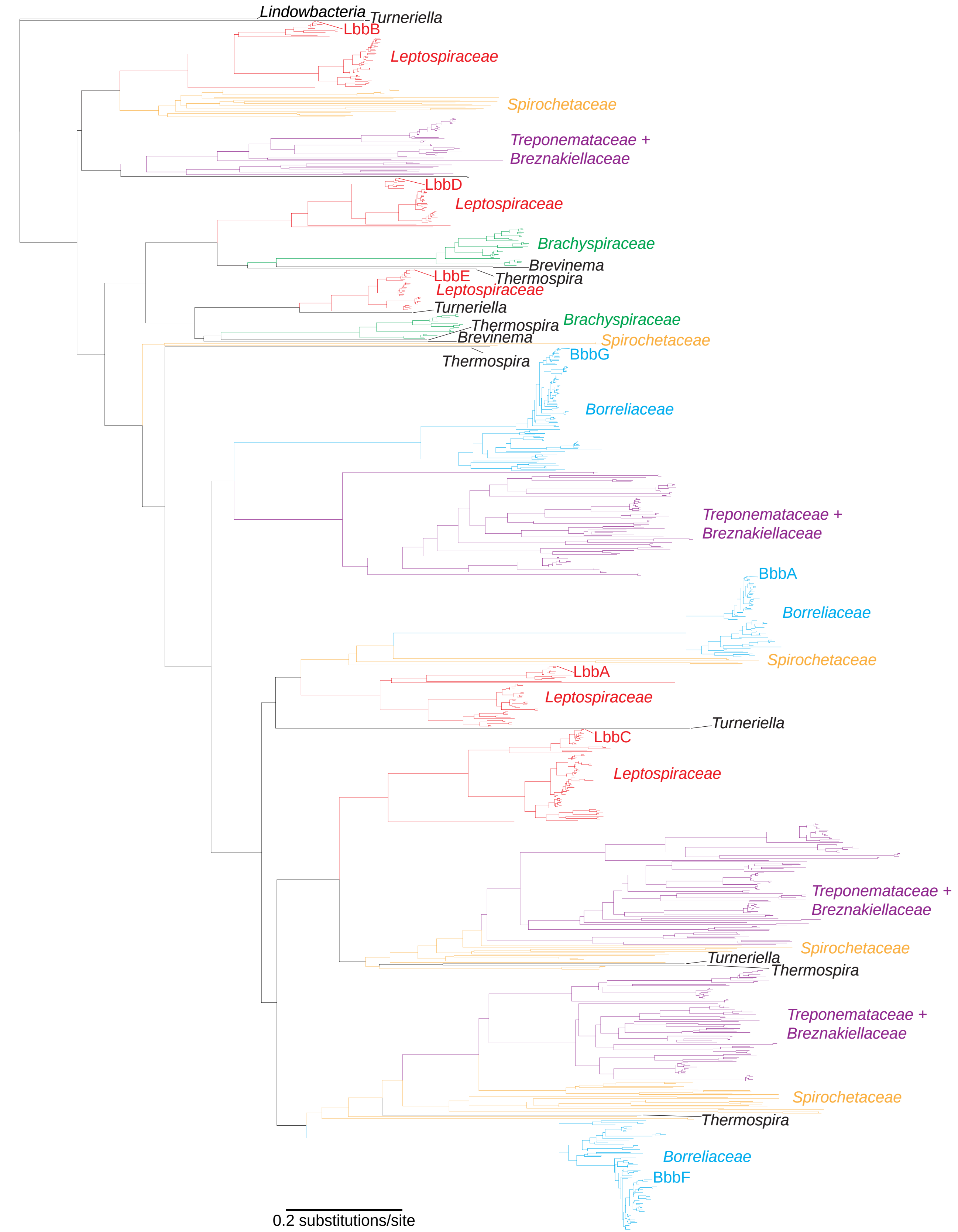

Lindowbacteria
Turneriella
LbbB
Leptospiraceae
Spirochetaceae
Treponemataceae +
Breznakiellaceae
LbbD
Leptospiraceae
Brachyspiraceae
Brevinema
LbbE
Thermospira
Leptospiraceae
Turneriella
Thermospira
Brachyspiraceae
Brevinema
Spirochetaceae
Thermospira
BbbG
Borreliaceae
Treponemataceae +
Breznakiellaceae
BbbA
Borreliaceae
Spirochetaceae
LbbA
Leptospiraceae
Turneriella
LbbC
Leptospiraceae
Treponemataceae +
Breznakiellaceae
Spirochetaceae
Turneriella
Thermospira
Treponemataceae +
Breznakiellaceae
Spirochetaceae
Thermospira
Borreliaceae
BbbF
0.2 substitutions/site

Figure S2

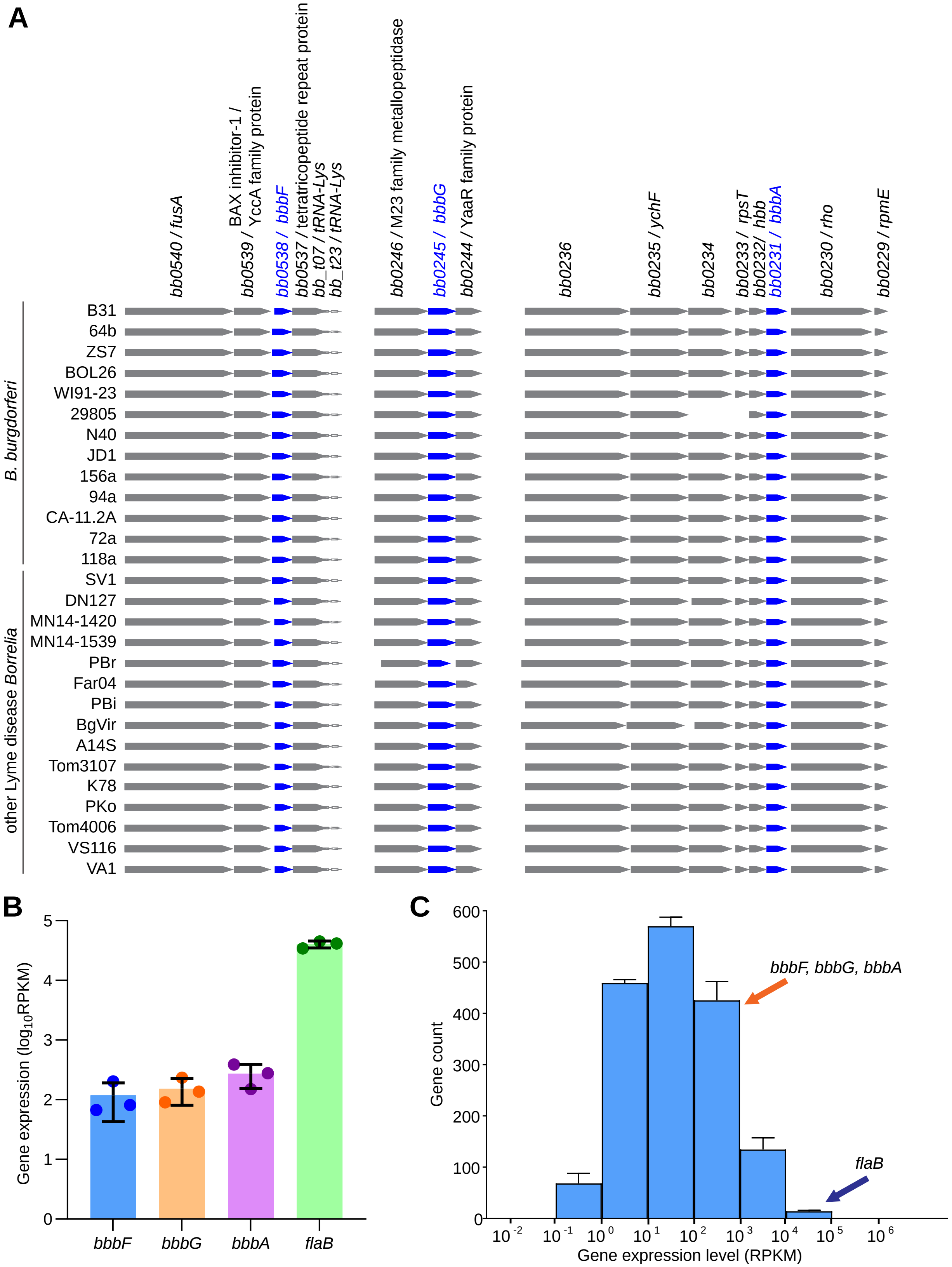

A
bb0540 / fusA
bb0539 / BAX inhibitor-1 / YccA family protein
bb0538 / bbbF
bb0537 / tetratricopeptide repeat protein
bb_t07 / tRNA-Lys
bb_t23 / tRNA-Lys
bb0246 / M23 family metallopeptidase
bb0245 / bbbG
bb0244 / YaaR family protein
bb0236
bb0235 / ychF
bb0234
bb0233 / rpsT
bb0232/ hbb
bb0231 / bbbA
bb0230 / rho
bb0229 / rpmE
B. burgdorferi
B31
64b
ZS7
BOL26
WI91-23
29805
N40
JD1
156a
94a
CA-11.2A
72a
118a
other Lyme disease Borrelia
SV1
DN127
MN14-1420
MN14-1539
PBr
Far04
PBi
BgVir
A14S
Tom3107
K78
PKo
Tom4006
VS116
VA1
B
Gene expression (log10RPKM)
0
1
2
3
4
5
bbbF
bbbG
bbbA
flaB
C
Gene count
0
100
200
300
400
500
600
bbbF, bbbG, bbbA
flaB
10 -2
10 -1
10 0
10 1
10 2
10 3
10 4
10 5
10 6
Gene expression level (RPKM)

Figure S3

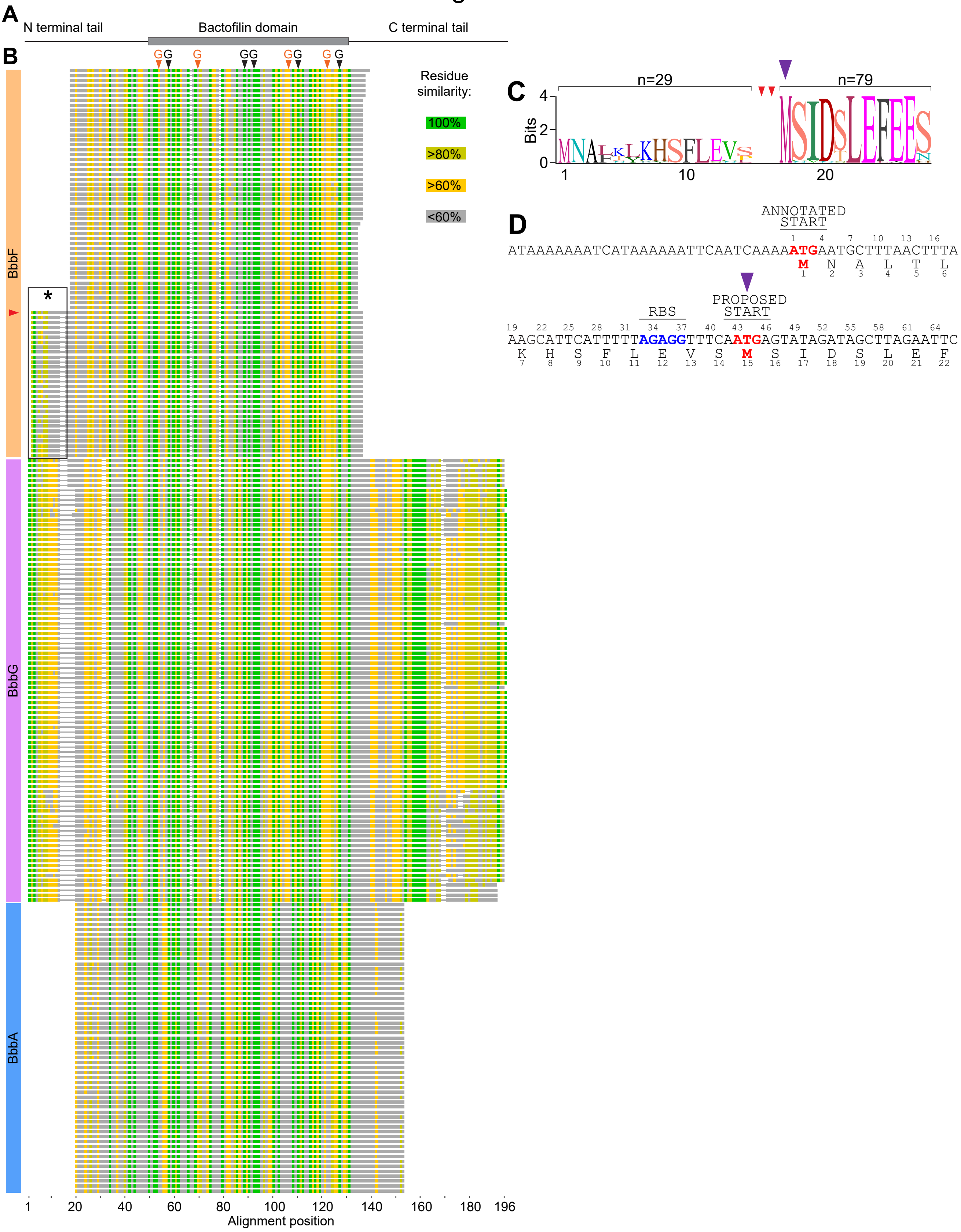

A
N terminal tail
Bactofilin domain
C terminal tail
B
GG G GG GG G G
Residue similarity:
100%
>80%
>60%
<60%
*
BbbF
BbbG
BbbA
1
20
40
60
80
100
120
140
160
180
196
Alignment position
C
n=29
n=79
4
2
0
Bits
MNALKLKHSFLEVS
MSIDSLEFEES
1
10
20
D
ANNOTATED START
1 4 7 10 13 16
ATAAAAAAATCATAAAAAATTCAATCAAAAATGAATGCTTTAACTTTA
M N A L T L
1 2 3 4 5 6
PROPOSED START
RBS
19 22 25 28 31 34 37 40 43 46 49 52 55 58 61 64
AAGCATTCATTTTTAGAGGTTTCAATGAGTATAGATAGCTTAGAATTC
K H S F L E V S M S I D S L E F
7 8 9 10 11 12 13 14 15 16 17 18 19 20 21 22

Figure S4

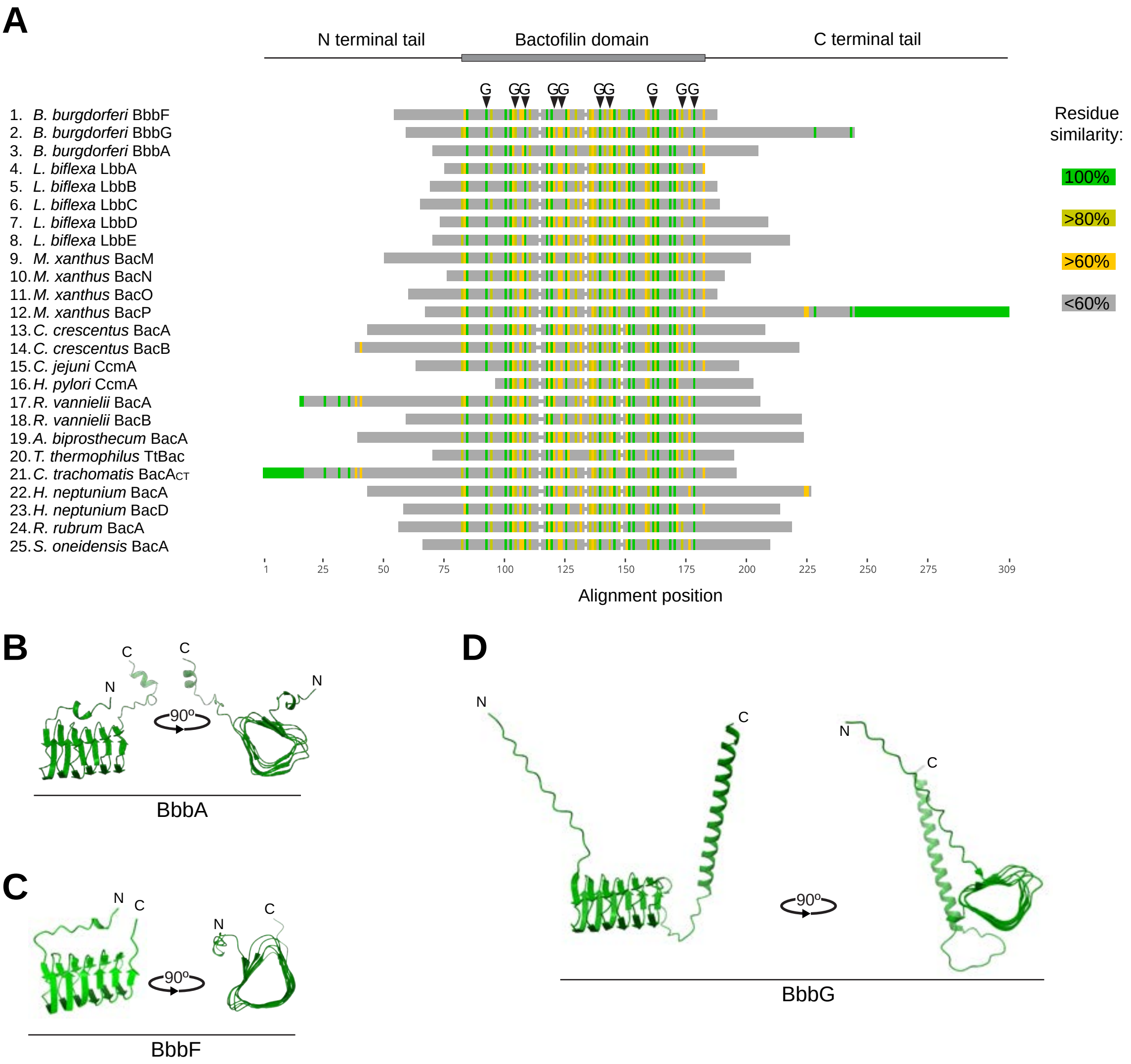

A
N terminal tail
Bactofilin domain
C terminal tail
G GG GG GG G GG
1. B. burgdorferi BbbF
2. B. burgdorferi BbbG
3. B. burgdorferi BbbA
4. L. biflexa LbbA
5. L. biflexa LbbB
6. L. biflexa LbbC
7. L. biflexa LbbD
8. L. biflexa LbbE
9. M. xanthus BacM
10. M. xanthus BacN
11. M. xanthus BacO
12. M. xanthus BacP
13. C. crescentus BacA
14. C. crescentus BacB
15. C. jejuni CcmA
16. H. pylori CcmA
17. R. vannielii BacA
18. R. vannielii BacB
19. A. biprosthecum BacA
20. T. thermophilus TtBac
21. C. trachomatis BacACT
22. H. neptunium BacA
23. H. neptunium BacD
24. R. rubrum BacA
25. S. oneidensis BacA
Residue similarity:
100%
>80%
>60%
<60%
1
25
50
75
100
125
150
175
200
225
250
275
309
Alignment position
B
C
C
N
N
90º
BbbA
C
N
C
N
C
90º
BbbF
D
N
C
N
C
90º
BbbG

Figure S5

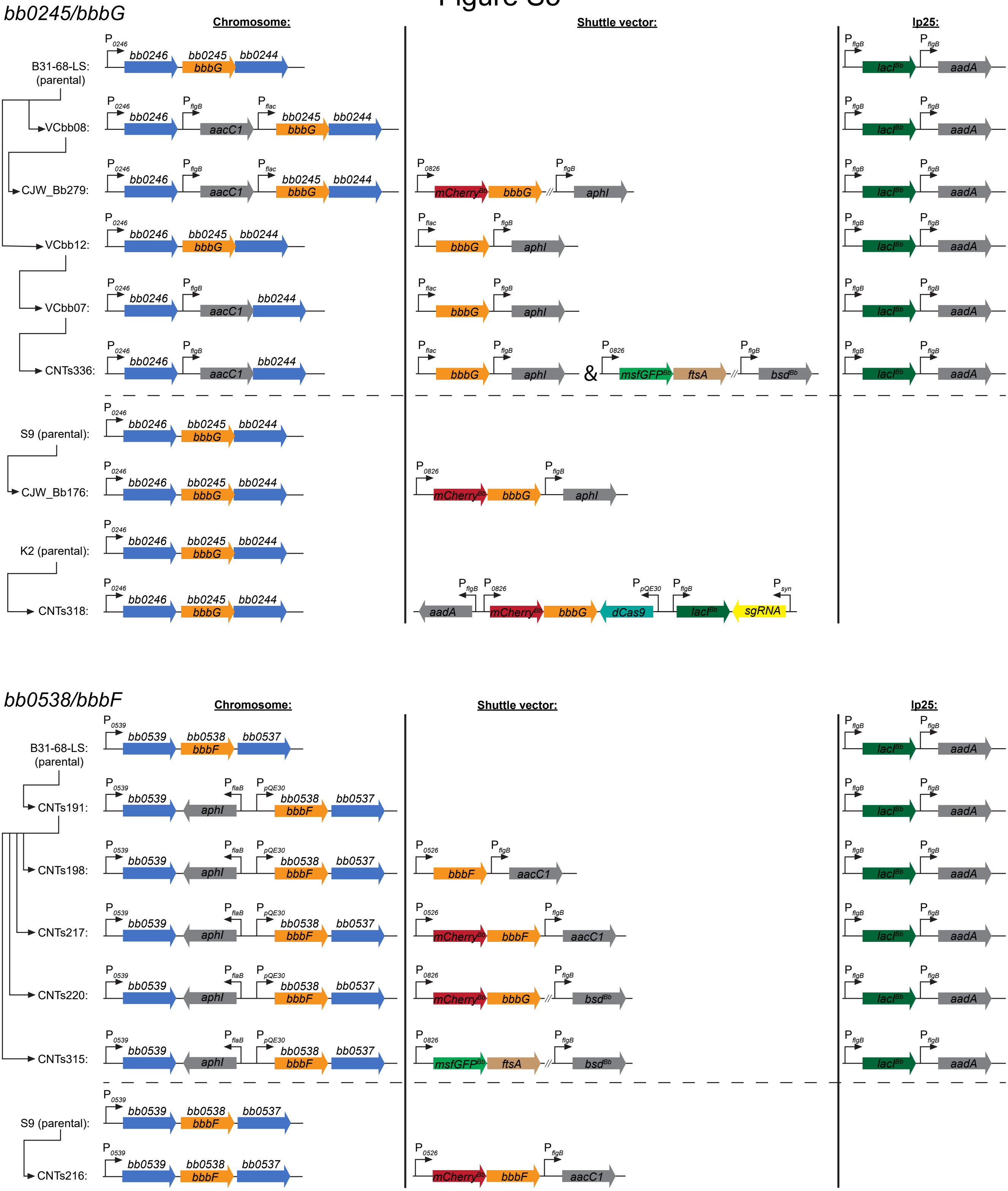
bb0245/bbbG
Chromosome:
Shuttle vector:
lp25:
B31-68-LS: (parental)
VCbb08:
CJW_Bb279:
VCbb12:
VCbb07:
CNTs336:
S9 (parental):
CJW_Bb176:
K2 (parental):
CNTs318:
bb0246
bb0245
bbbG
bb0244
aacC1
mCherry
aphI
msfGFP
ftsA
bsd
aadA
dCas9
lacI
sgRNA
bb0538/bbbF
Chromosome:
Shuttle vector:
lp25:
B31-68-LS: (parental)
CNTs191:
CNTs198:
CNTs217:
CNTs220:
CNTs315:
S9 (parental):
CNTs216:
bb0539
bb0538
bbbF
bb0537

Figure S6

A

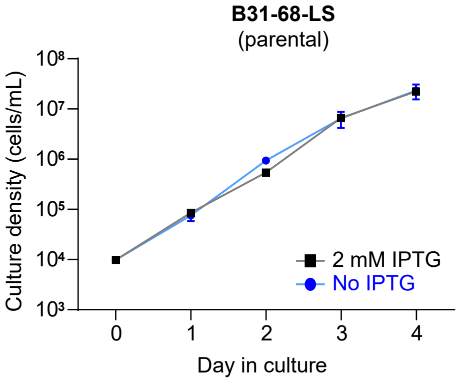

B

```
                 298
Parental lacI    GAACGAAGCGGCGTCGAAGCC
                  E  R  S  G  V  E  A
                 100

                 298
Mutant lacI      GAACGAAGCGGCGTCTAAGCC
                  E  R  S  G  V  *  A
                 100
```

C

```
                              lacO                  RBS          START
Pflac     TAATTTAGTTAAAATTGTGAGCGCTCACAATTGAGGGAGGTTTCCATATG
mutPflac  TAATTTAGTTAAAA      CGTTTTATGTAATTGAGGGAGGTTTCCATATG
```

Figure S7

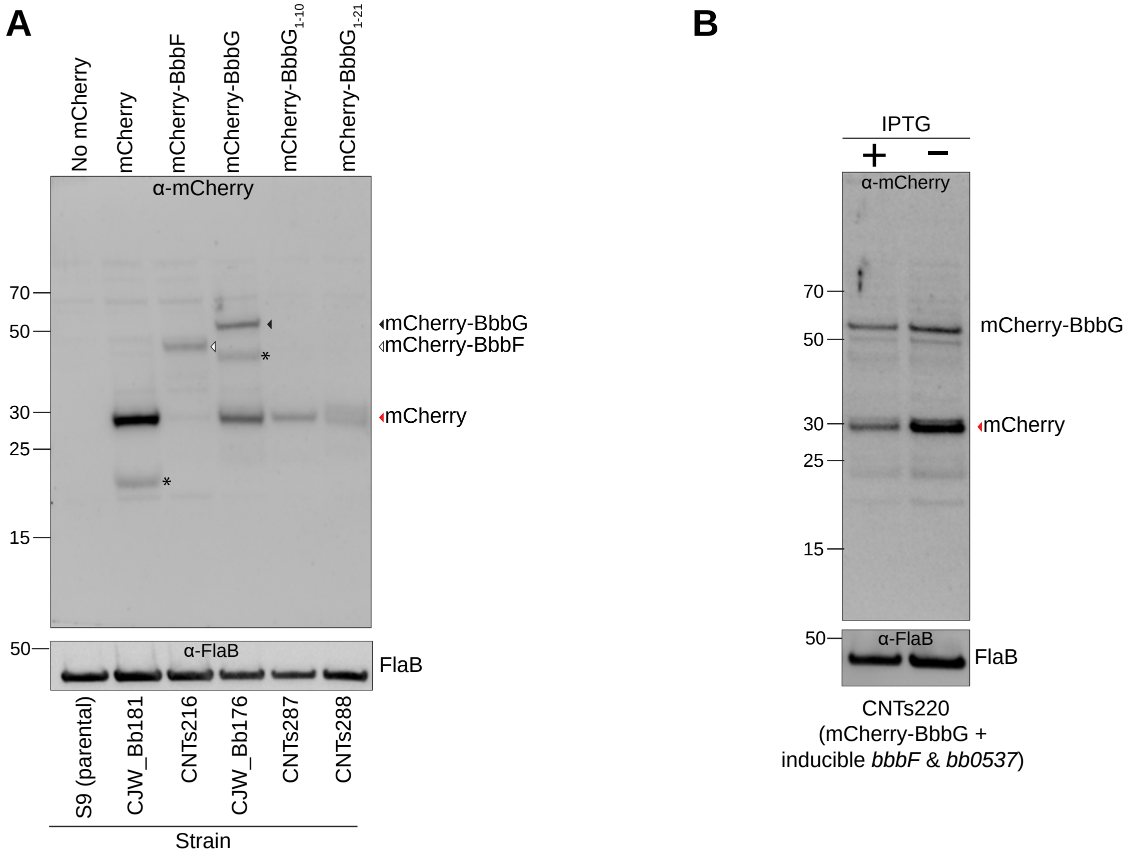
A
No mCherry
mCherry
mCherry-BbbF
mCherry-BbbG
mCherry-BbbG1-10
mCherry-BbbG1-21
α-mCherry
70
50
30
25
15
mCherry-BbbG
mCherry-BbbF
mCherry
*
*
50
α-FlaB
FlaB
S9 (parental)
CJW_Bb181
CNTs216
CJW_Bb176
CNTs287
CNTs288
Strain
B
IPTG
+
−
α-mCherry
70
50
30
25
15
mCherry-BbbG
mCherry
50
α-FlaB
FlaB
CNTs220
(mCherry-BbbG +
inducible bbbF & bb0537)

Figure S8

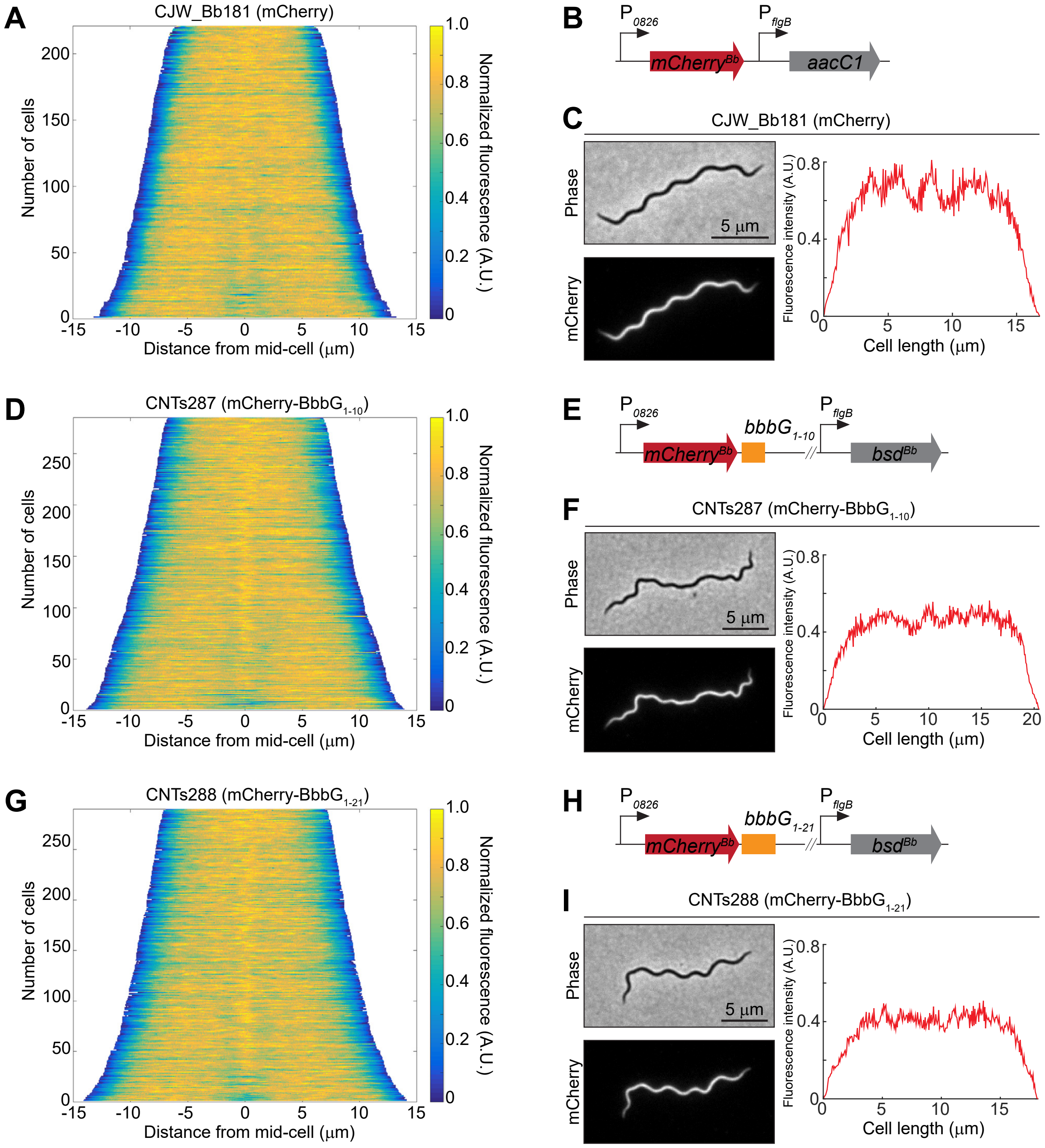

A
CJW_Bb181 (mCherry)
Number of cells
Distance from mid-cell (μm)
Normalized fluorescence (A.U.)
B
P0826
PflgB
mCherryBb
aacC1
C
CJW_Bb181 (mCherry)
Phase
mCherry
5 μm
Fluorescence intensity (A.U.)
Cell length (μm)
D
CNTs287 (mCherry-BbbG1-10)
Number of cells
Distance from mid-cell (μm)
Normalized fluorescence (A.U.)
E
P0826
bbbG1-10
PflgB
mCherryBb
bsdBb
F
CNTs287 (mCherry-BbbG1-10)
Phase
mCherry
5 μm
Fluorescence intensity (A.U.)
Cell length (μm)
G
CNTs288 (mCherry-BbbG1-21)
Number of cells
Distance from mid-cell (μm)
Normalized fluorescence (A.U.)
H
P0826
bbbG1-21
PflgB
mCherryBb
bsdBb
I
CNTs288 (mCherry-BbbG1-21)
Phase
mCherry
5 μm
Fluorescence intensity (A.U.)
Cell length (μm)

Figure S9

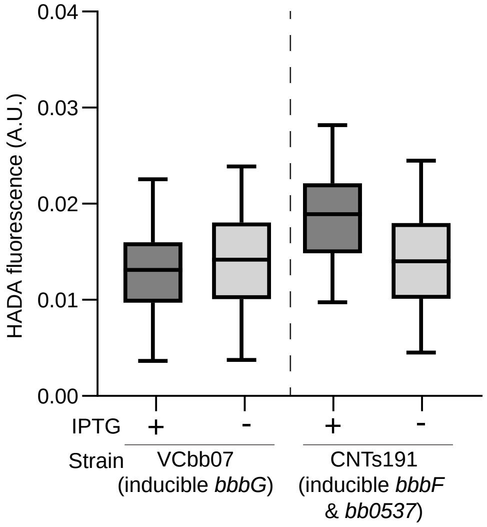
0.04
0.03
0.02
0.01
0.00
HADA fluorescence (A.U.)
IPTG
+
-
+
-
Strain
VCbb07
(inducible *bbbG*)
CNTs191
(inducible *bbbF* & *bb0537*)

Figure S10

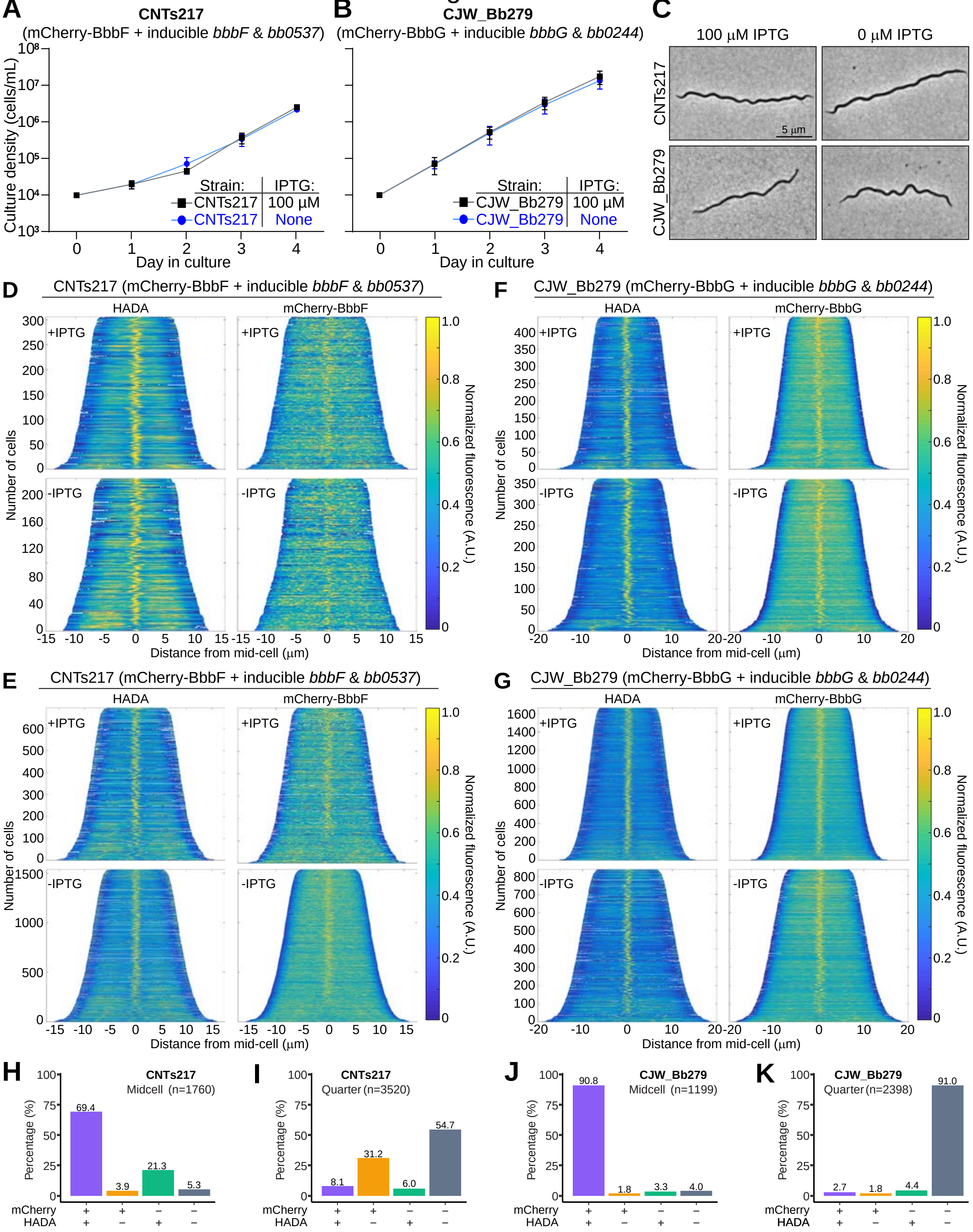

A
CNTs217
(mCherry-BbbF + inducible bbbF & bb0537)
Culture density (cells/mL)
Day in culture
Strain: IPTG:
CNTs217 100 µM
CNTs217 None
B
CJW_Bb279
(mCherry-BbbG + inducible bbbG & bb0244)
Strain: IPTG:
CJW_Bb279 100 µM
CJW_Bb279 None
C
100 µM IPTG
0 µM IPTG
CNTs217
CJW_Bb279
5 µm
D
CNTs217 (mCherry-BbbF + inducible bbbF & bb0537)
HADA
mCherry-BbbF
+IPTG
-IPTG
Number of cells
Normalized fluorescence (A.U.)
Distance from mid-cell (µm)
F
CJW_Bb279 (mCherry-BbbG + inducible bbbG & bb0244)
HADA
mCherry-BbbG
E
CNTs217 (mCherry-BbbF + inducible bbbF & bb0537)
G
CJW_Bb279 (mCherry-BbbG + inducible bbbG & bb0244)
H
CNTs217
Midcell (n=1760)
Percentage (%)
69.4
3.9
21.3
5.3
I
CNTs217
Quarter (n=3520)
8.1
31.2
6.0
54.7
J
CJW_Bb279
Midcell (n=1199)
90.8
1.8
3.3
4.0
K
CJW_Bb279
Quarter (n=2398)
2.7
1.8
4.4
91.0
mCherry + + – –
HADA + – + –

Figure S11

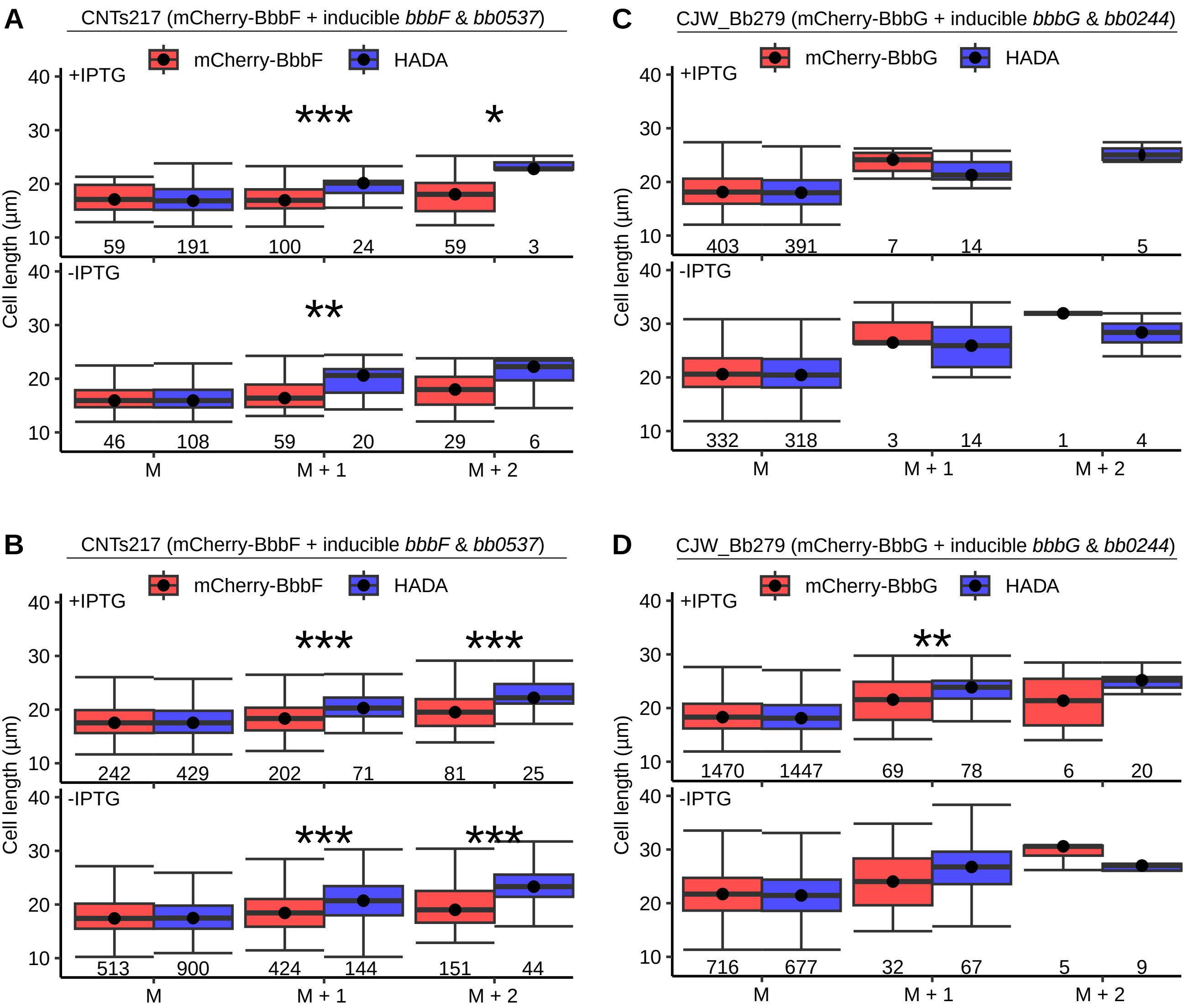
A
CNTs217 (mCherry-BbbF + inducible bbbF & bb0537)
mCherry-BbbF
HADA
+IPTG
-IPTG
Cell length (µm)
***
*
**
59
191
100
24
59
3
46
108
59
20
29
6
M
M + 1
M + 2
C
CJW_Bb279 (mCherry-BbbG + inducible bbbG & bb0244)
mCherry-BbbG
HADA
403
391
7
14
5
332
318
3
14
1
4
B
CNTs217 (mCherry-BbbF + inducible bbbF & bb0537)
***
***
***
***
242
429
202
71
81
25
513
900
424
144
151
44
D
CJW_Bb279 (mCherry-BbbG + inducible bbbG & bb0244)
**
1470
1447
69
78
6
20
716
677
32
67
5
9

Figure S12

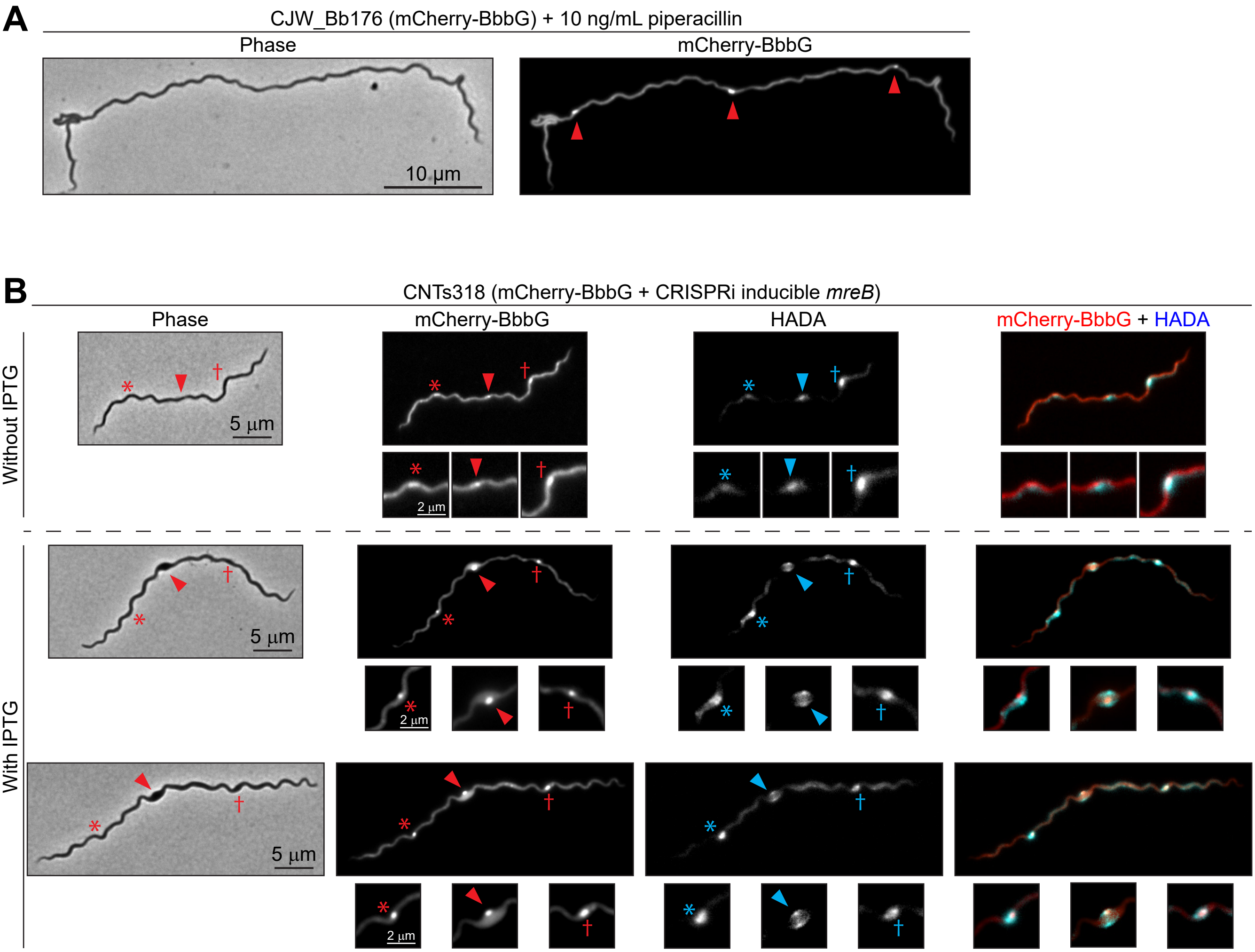
A
CJW_Bb176 (mCherry-BbbG) + 10 ng/mL piperacillin
Phase
mCherry-BbbG
10 µm
B
CNTs318 (mCherry-BbbG + CRISPRi inducible *mreB*)
Phase
mCherry-BbbG
HADA
mCherry-BbbG + HADA
Without IPTG
With IPTG
5 µm
2 µm
