# Supplementary material for: Bactofilins are essential spatial organizers of peptidoglycan insertion in the Lyme disease spirochete Borrelia burgdorferi
Source: J Bacteriol. 2026 Jun 29;208(7):e00198-26. doi: 10.1128/jb.00198-26 (PMC13393453; doi:10.1128/jb.00198-26)
Supplement: Supplemental legends — Legends for Figures S1 to S12 and Tables S1 to S9. [file jb.00198-26-s0002.docx]

**Supplementary materials**

**SUPPLEMENTARY FIGURE LEGENDS**

**Figure S1. Phylogenetic tree of bactofilin sequences encoded by spirochetes.**

The tree was generated using 879 non-redundant spirochete bactofilin sequences rooted on *Lindowbacteria*. Colored branches indicate bactofilin clades belonging to the listed families. Black branches and names indicate genera with low sequence representation. The five *Leptospira biflexa* proteins LbbA through LbbE are indicated on the tree. The three bactofilins encoded by *B. burgdorferi* strain B31, BbbA, BbbF, and BbbG, are also indicated.

**Figure S2. Genomic context and native expression of the *B. burgdorferi* bactofilins.**

**A.** Bactofilin loci in 28 Lyme disease *Borrelia* genomes visualized using the BorreliaBase online resource (1). Strain names are listed on the left. Gene numbers listed at the top are based on the genome of the *B. burgdorferi* type strain B31. Bactofilin-encoding genes are shown in blue.

**B.** Bactofilin gene expression detected by RNA-seq in strain B31-68-LS during in vitro cultivation. The highly expressed *flaB* gene is included for reference. Shown are individual values as well as means ± standard deviations of *n*=3 replicates. The RNA-seq data was published in a previous study (2). RPKM, reads per kilobase per million reads.

**C.** Histogram depicting the expression of all the genes detected in strain B31-68-LS during in vitro cultivation. The genes are grouped in successive 10-fold expression bins from 10^-2^ to 10^6^ RPKM. Each bar depicts the mean number of genes that belong to each expression bin, while the error bars depict the standard deviation of this number for the *n*=3 replicates analyzed. The bin containing the three bactofilin genes *bbbA*, *bbbF*, and *bbbG* is indicated by an orange arrow. For reference, the bin containing the highly expressed *flaB* gene is indicated by a blue arrow.

**Figure S3. Alignment of *Borreliaceae* bactofilin sequences.**

**A.** Schematic depiction of domain organization of *B. burgdorferi* bactofilins, matching the sequence alignment shown in panel B.

**B.** Sequence alignment of 232 bactofilin sequences encoded by *Borreliaceae*. The three clades, BbbF, BbbG, and BbbA, are indicated at the left. A subset of BbbF sequences have an extended N-terminal tail, marked by a star (*) sign. A single BbbF sequence with two additional residues in the extended N-terminal tail is marked by a red arrowhead. Color map for residue similarity levels are at the right. Conserved glycine (G) residues all bactofilins are indicated at the top in black. Glycine residues conserved in BbbF and BbbG but not BbbA are indicated at the top in orange.

**C.** Sequence logo of 79 *B. burgdorferi* BbbF sequences. Some of these sequences (*n*=29) have an N-terminal tail extension of 14 amino acids. One sequence has an N-terminal tail extension of 16 amino acids leading to a two-residue gap in the logo (red arrowheads). The methionine at position 17 in the alignment (position 15 in 78 out of 79 sequences) indicated by a purple arrowhead is a conserved START site in all 79 sequences.

**D.** Nucleotide sequence of the *B. burgdorferi* B31 genome (3) near the beginning of the *bbbF* gene and its encoded protein sequence. Nucleotide bases are listed from left to right in the 5’ to 3’ direction of the coding strand of *bbbF*. Putative start codons are in red, with the leftmost being the annotated start codon. Encoded amino acids are listed below the nucleotide sequence using the single letter convention. Likely ribosomal binding site (RBS) is in blue. Functional start codon used in this study is indicated by the same arrowhead as in (A). Nucleotide numbering is listed above the sequence, starting at the first nucleotide of the annotated start codon. Amino-acid numbering is listed below the sequence.

**Figure S4. Sequence alignment and structural prediction *B. burgdorferi* bactofilin sequences.**

**A.** Sequence alignment of bactofilins that have been investigated experimentally, as well as the four additional *L. biflexa* bactofilin sequences, and three *B. burgdorferi* bactofilins. Schematic depiction of domain organization of bactofilins is at the top. Conserved glycine residues are indicated by G’s at the top of the alignment. Color maps for residue similarity levels are at the right.

**B.** Alphafold3 structural prediction model of bactofilin BbbA encoded by *B. burgdorferi* strain B31. Amino (N) and carboxy (C) termini of the polypeptide chains are indicated. Shown are views from the side of the bactofilin domain (left) and along the central axis of the bactofilin domain (right).

**C.** Same as in B but for bactofilin BbbF of *B. burgdorferi* strain B31. Only residues between the annotated M15 position and the end of the protein were modeled.

**D.** Same as in B but for bactofilin BbbG of *B. burgdorferi* strain B31.

**Figure S5. Schematic depictions of relevant gene features of the strains used in this study.**

Relevant features of strains generated to investigate BbbG are at the top, while those of strains generated to investigate BbbF are at the bottom. The chromosomal loci are depicted in the left column. If present, relevant genes expressed from shuttle vectors are listed in the middle column. For strains derived from B31-68-LS, l*acI^Bb^* expression from endogenous plasmid lp25 is shown in the right column. Arrows at the left trace strain lineages.

**Figure S6. Characterization of parental strain B31-68-LS and bactofilin expression knockdown escape mutants.**

**A.** Growth curves of parental strain B31-68-LS grown with 2 mM IPTG or no IPTG. Shown are means ± standard deviations of results from *n* = 3 cultures.

**B.** Comparison of *lacI* sequences from VCbb08 and its escape mutant highlighting a single-nucleotide substitution at position 313 (red) that generated a premature stop codon (purple arrowhead) and can cause expression of a truncated LacI protein missing 70% of its sequence.

**C.** Comparison of the *P_flac_* sequences from VCbb07 and its escape mutant revealing extensive mutations (red) within the *lacO* sequence.

**Figure S7. Western blotting detection of mCherry-bactofilin fusions**

**A.** Western blotting of lysates of strains used in this study. Top, blotting with an anti-mCherry antibody. Bottom, blotting with an anti-FlaB antibody of the same membrane used for the anti-mCherry blot. Identities of the major bands are given at the right, indicated by corresponding arrows. Protein size standards (kDa) are indicated at the left. Strain identities are at the bottom. Relevant expressed proteins are at the top. Bands marked by an asterisk are consistent with mCherry degradation products that can be generated during protein boiling prior to SDS-PAGE (4). Bands corresponding to free mCherry or products of length indistinguishable from it are indicated by the red arrow.

**B.** Western blotting of lysates of strain CNTs220 grown with 1 mM IPTG or without IPTG for 24 h. Top, blotting with an anti-mCherry antibody. Bottom, blotting with anti-FlaB antibody of the same membrane used for the anti-mCherry blot. Bands corresponding to free mCherry or products of length indistinguishable from it are indicated by the red arrow.

**Figure S8. Localization of free mCherry and mCherry fused to BbbG N-terminal fragments**

**A.** Demograph of a population of 221 cells of strain CJW_Bb181 producing free mCherry. A.U., arbitrary units.

**B.** Schematic depiction of the relevant plasmid features for strain CJW_Bb181.

**C.** Phase contrast and fluorescence micrographs (left), and fluorescence intensity profile (right) of a cell of strain CJW_Bb181 producing free mCherry. A.U., arbitrary units.

**D.** Same as in A but for a population of 285 cells of strain CNTs287 producing mCherry-BbbG_1-10_.

**E.** Same as in B but for strain CNTs287

**F.** Same as in C but for a cell of strain CNTs287.

**G.** Same as in A but for a population of 290 cells of strain CNTs288 producing mCherry-BbbG_1-21_.

**H.** Same as in B but for strain CNTs288

**I.** Same as in C but for a cell of strain CNTs288.

**Figure S9. Quantification of HADA uptake by bactofilin expression knockdown strains.**

Total HADA fluorescence normalized by cell area for populations of cells of strain VCbb07 (carrying IPTG-inducible *bbbG*, left) or strain CNTs191 (carrying IPTG-inducible *bbbF* and *bb0537*, right) grown in the presence or absence of 100 µM IPTG. Cell populations are the same used in the demographs in Figs. 3G, 3I, 3A, and 3C respectively). Boxes represent the interquartile ranges of cell fluorescence with the mean as the midline; tails represent the 2.5 and 97.5 percentiles. A.U., arbitrary units.

**Figure S10. Complementation of bactofilin expression knockdown by mCherry-bactofilin fusions**

**A.** Growth curve of strain CNTs217 (carrying IPTG-inducible *bbbF* and *bb0537* along with shuttle vector-borne, constitutively expressed *mcherry^Bb^-bbbF*) grown in the presence or absence of 100 µM IPTG. Points are means ± standard deviations of results from *n* = 3 cultures.

**B.**  Growth curve of strain CJW_Bb279 (carrying IPTG-inducible *bbbG* and *bb0244* along with shuttle-vector borne, constitutively expressed *mcherry^Bb^-bbbG*) grown in the presence or absence of 100 µM IPTG. Points are means ± standard deviations of results from *n* = 3 cultures.

**C.** Phase contrast light micrographs of cells of strains CNTs217 (top) and CJW_Bb279 (bottom) grown with 100 μM IPTG (left) or for 48 hours without IPTG (0 μM IPTG, right).

**D** and **E.** Demographs of populations of cells of strain CNTs217 grown with 100 μM IPTG (top) or grown in the absence of IPTG for 24 hours (bottom). The cells were labeled with HADA for 60 min. Demographs of the HADA signal are on the left, while those of the mCherry-BbbF signal are on the right. The cells are arrayed from the shortest at the top to the longest at the bottom and are aligned along their mid-cell positions. The color of each line reflects the distribution of the fluorescent signal along the cell length, according to the color scale at the right. A.U., arbitrary units. Panel D shows data from one experiment (replicate 1, top, +IPTG, *n =* 306 cells, and bottom, -IPTG, *n =* 223 cells), while panel E shows data from the second experiment (replicate 2, top, +IPTG, *n =* 695 cells, and bottom, -IPTG, *n =* 1,537 cells).

**F** and **G.** Demographs of populations of cells of strain CJW_Bb279 grown with 100 μM IPTG (top) or grown in the absence of IPTG for 24 hours (bottom). The cells were labeled with HADA for 60 min. Demographs of the HADA signal are on the left, while those of the mCherry-BbbG signal are on the right. The cells are arrayed from the shortest at the top to the longest at the bottom and are aligned along their mid-cell positions. The color of each line reflects the distribution of the fluorescent signal along the cell length, according to the color scale at the right. A.U., arbitrary units. Panel F shows data from one experiment (replicate 1, top, +IPTG, *n =* 445 cells, and bottom, -IPTG, *n =* 362 cells), while panel G shows data from the second experiment (replicate 2, top, +IPTG, *n =* 1,660 cells, and bottom, -IPTG, *n =* 837 cells).

**H.** Bar graph quantifying the presence of mid-cell mCherry-BbbF and/or HADA peaks in a population of 1,760 cells of strain CNTs217 grown without IPTG for 24 hours and labeled with HADA. Mid-cell signal peaks were defined using a fluorescent spot finder algorithm restricted to a cell region centered at mid-cell and corresponding to 15% of cell length. From left to right, the bars indicate the fraction of mid-cell regions containing both an mCherry-BbbF and a HADA peak, only an mCherry-BbbF peak, only a HADA peak, and no peak in either of the signals, respectively.

**I.** Same as in H but quantifying the presence of mCherry-BbbF and/or HADA peaks in the one-quarter and three-quarter cell regions (*n* = 3,520 quarter-cell regions in 1,760 cells). Quarter-cell signal peaks were defined using a fluorescent spot finder algorithm restricted to the cell regions centered at the one-quarter and three-quarter cell positions and corresponding to 15% of cell length.

**J.** Bar graph quantifying the presence of mid-cell mCherry-BbbG and/or HADA peaks in a population of 1,199 cells of strain CJW_Bb279 grown without IPTG for 24 hours and labeled with HADA. Mid-cell signal peaks were defined using a fluorescent spot finder algorithm restricted to a cell region centered at mid-cell and corresponding to 15% of cell length. From left to right, the bars indicate the fraction of mid-cell regions containing both an mCherry-BbbG and a HADA peak, only an mCherry-BbbG peak, only a HADA peak, and no peak in either of the signals, respectively.

**K.** Same as in J but quantifying the presence of mCherry-BbbG and/or HADA peaks in the one-quarter and three-quarter cell regions (*n* = 2,398 quarter-cell regions in 1,199 cells). Quarter-cell signal peaks were defined using a fluorescent spot finder algorithm restricted to the cell regions centered at the one-quarter and three-quarter cell positions and corresponding to 15% of cell length.

**Figure S11. Comparison of cell length distributions in subpopulations of cells sorted based on zonal localization of mCherry-bactofilin or HADA signals.**

**A.** Boxplots depicting the length distributions of cells of strain CNTs217 grown with (top, *n=*218 cells) or without (bottom, *n=*134 cells) 100 μM IPTG and labeled with HADA. This is experimental replicate 1 included in Fig. 5F. Cells were grouped based on the mCherry-BbbF signal (in red) or the HADA signal (in blue) into the M, M+1, and M+2 classes as defined in Fig. 5E.

**B.** Same as in A. but for replicate 2; top, with IPTG, *n=*525; bottom, without IPTG, *n=*1088.

**C.** Boxplots depicting the length distributions of cells of strain CJW_Bb279 grown with (top, *n=*410 cells) or without (bottom, *n=*336 cells) 100 μM IPTG and labeled with HADA. This is experimental replicate 1 included in Fig. 5G. Cells were grouped based on the mCherry-BbbG signal (in red) or the HADA signal (in blue) into the M, M+1, and M+2 classes as defined in Fig. 5E.

**D.** Same as in C. but for replicate 2; top, with IPTG, *n=*1545; bottom, without IPTG, *n=*753.

**A-D.** For each signal and class, analyzed cell numbers are shown above the x-axis. Boxes represent the interquartile ranges of cell lengths with the median as the midline; tails represent the 2.5 and 97.5 percentiles. The two-tailed, paired Wilcoxon test was used to measure significance of the differences in median cell length within each growth stage between the grouping based on the mCherry-bactofilin signal and that based on the HADA signal. *p*-values are shown above the box plots: ***, *p*<0.0001; **, *p*<0.001; *, *p*<0.01.

**Figure S12. Effects of FtsI inhibition and *mreB* expression knockdown on the localization of mCherry-BbbG**

**A.** Phase contrast (left) and fluorescence (right) micrographs of a cell of strain CJW_Bb176 (expressing *mCherry^Bb^-bbbG*) grown with 10 ng/mL piperacillin for 24 hours. Red arrows indicate sites of mCherry-BbbG accumulation.

**B.** Cells of strain CNTs318 (constitutively expressing *mCherry^Bb^-bbbG* and carrying IPTG-inducible CRISPR interference constructs targeting *mreB*) grown in the absence of IPTG and thus expressing *mreB*, or cultured for 24 hours with 1 mM IPTG. From left to right, the images are: phase contrast micrograph; fluorescence micrograph of mCherry-BbbG; fluorescence micrograph of HADA; and overlayed image of the mCherry-BbbG signal (in red) with the HADA signal (in cyan). Insets are zoomed images of the mid-cell (denoted by an arrowhead), as well as one-quarter and three-quarter positions along the cell length, (denoted by an asterisk and cross, respectively).

**SUPPLEMENTARY TABLE LEGENDS**

**Table S1. Oufti segmentation parameters used in this study.** For each segmentation run done in this study, the segmentation parameters used are shown. Parameters are listed with the strains, imaging conditions, and relevant figures that use the segmented cell data.

**Table S2. Jonckheere-Terpstra IPTG growth condition and replicate test summaries.** For each permutation of strain, growth in the presence or absence of 100 µM IPTG, and replicate, the number of cells per signal category (including other) are shown. Significant Jonckheere-Terpstra p-values indicate a positive increase in length by increasing stage (i.e., M, M+1, and M+2): ***, *p*<0.0001.

**Table S3. Jonckheere-Terpstra test summaries for cells grown in the presence of 100 µM IPTG.** For strains CNTs217, CJW_Bb279, CNTs315, and CNTs336 grown with 100 µM IPTG, the number of cells per signal category (including other) are shown. The counts for strains CNTs217 and CJW_Bb279 are the combined values for replicate 1 and 2 in Table S2. Significant Jonckheere-Terpstra p-values indicate a positive increase in length by increasing stage (i.e., M, M+1, and M+2): ***, *p*<0.0001; **, *p*<0.001.

**Table S4. Order of arrival tests by Wilcoxon rank sum for each bactofilin strain condition**. For each permutation of strain, growth in the presence or absence of IPTG, replicate, and signal stage, a Wilcoxon rank sum comparison was done to compare median cell length between the two signal categories. Significant p-values indicate a difference between the signal median lengths: ***, *p*<0.0001; **, *p*<0.001; *, *p*<0.01, and cases where the bactofilin signal is detected in significantly shorter cells are indicated as “TRUE”. Replicate 1 of strain CJW_Bb279 grown with IPTG had no cells in the M+2 category as sorted using the mCherry-BbbG signal, which precluded us from performing the analysis.

**Table S5. Order of arrival tests by Wilcoxon for cells grown in the presence of 100 µM IPTG**. For strains CNTs217, CJW_Bb279, CNTs315, and CNTs336 grown with 100 µM IPTG, a Wilcoxon rank sum comparison was done to compare median cell length between the two signal categories. The counts for strains CNTs217 and CJW_Bb279 are the combined values for replicate 1 and 2 in Table S2. Significant p-values indicate a difference between the signal median lengths: ***, *p*<0.0001; **, *p*<0.001; *, *p*<0.01, and cases where the bactofilin or FtsA signal is detected in significantly shorter cells are indicated as “TRUE”.

**Table S6. mCherry-bactofilin and HADA peak detection results for cells of strains CNTs217 and CJW_Bb279.** For each analyzed cell of strain CNTs217 and CJW_Bb279, the mCherry-bactofilin and HADA peak detection results at mid-cell, one-quarter, and three-quarter cell positions are indicated as either present (1) or absent (0). Cells are further identified by the bactofilin under control of an IPTG inducible promoter (Bactofilin = BB0245 [*bbbG*] or BB0538 [*bbbF*]), their replicate (Run = First or Second), presence of 100 µM IPTG in the growth media (IPTG = Positive or Negative), and their CellIndex and CellID labels which correspond to the frame and individual mesh identity number in the corresponding Oufti-generated mesh file. Total cell length in µm is also included.

**Table S7. msfGFP-FtsA and HADA peak detection results for strains CNTs315 and CNTs336.** For each cell analyzed cell of strain CNTs315 and CNTs336, the msfGFP-FtsA and HADA peak detection results at mid-cell, one-quarter, and three-quarter cell positions are indicated as either present (1) or absent (0). All cells were grown in the presence of 100 µM IPTG (IPTG = Positive). Cells are further identified by the bactofilin under control of an IPTG inducible promoter (Bactofilin = BB0245 [*bbbG*] or BB0538 [*bbbF*]). CellIndex and CellID labels correspond to the frame and individual mesh identity number in the corresponding Oufti-generated mesh file. Total cell length in µm is also included.

**Table S8. Population-level quantification of mCherry-bactofilin and HADA signal peak occurrences at mid-cell and quarter-cell positions in cells of strains CJW_Bb279 and CNTs217 grown with 100 µM IPTG.** Each cell listed in Table S6 and derived from a culture grown with 100 µM IPTG was classified by the presence (1) or absence (0) of mCherry-bactofilin and HADA signal peaks at mid-cell or at the one-quarter and three-quarter cell positions (labeled as Quarter). The “Protein” column indicates which bactofilin is under IPTG-inducible control. The “Count” column represents the number of cells classified based on the presence of mid-cell signal peaks in the two channels, or the number of quarter-cell positions classified into the same categories. The total number of quarter-cell positions is twice the number of cells or mid-cell positions.

**Table S9. Population-level quantification of mCherry-bactofilin and HADA signal peak occurrences at mid-cell and quarter-cell positions in cells of strains CJW_Bb279 and CNTs217 grown in the absence of IPTG.** Each cell listed in Table S6 and derived from a culture grown in the absence of IPTG was classified by the presence (1) or absence (0) of mCherry-bactofilin and HADA signal peaks at mid-cell or at the one-quarter and three-quarter cell positions (labeled as Quarter). The “Protein” column indicates which bactofilin is under IPTG-inducible control. The “Count” column represents the number of cells classified based on the presence of mid-cell signal peaks in the two channels, or the number of quarter-cell positions classified into the same categories. The total number of quarter-cell positions is twice the number of cells or mid-cell positions.

**SUPPLEMENTAL REFERENCES**

1. Di L, Pagan PE, Packer D, Martin CL, Akther S, Ramrattan G, Mongodin EF, Fraser CM, Schutzer SE, Luft BJ, Casjens SR, Qiu WG. 2014. BorreliaBase: a phylogeny-centered browser of *Borrelia* genomes. BMC Bioinformatics 15:233.

2. Wachter J, Cheff B, Hillman C, Carracoi V, Dorward DW, Martens C, Barbian K, Nardone G, Renee Olano L, Kinnersley M, Secor PR, Rosa PA. 2023. Coupled induction of prophage and virulence factors during tick transmission of the Lyme disease spirochete. Nat Commun 14:198.

3. Fraser CM, Casjens S, Huang WM, Sutton GG, Clayton R, Lathigra R, White O, Ketchum KA, Dodson R, Hickey EK, Gwinn M, Dougherty B, Tomb JF, Fleischmann RD, Richardson D, Peterson J, Kerlavage AR, Quackenbush J, Salzberg S, Hanson M, van Vugt R, Palmer N, Adams MD, Gocayne J, Weidman J, Utterback T, Watthey L, McDonald L, Artiach P, Bowman C, Garland S, Fuji C, Cotton MD, Horst K, Roberts K, Hatch B, Smith HO, Venter JC. 1997. Genomic sequence of a Lyme disease spirochaete, *Borrelia burgdorferi*. Nature 390:580-6.

4. Gross LA, Baird GS, Hoffman RC, Baldridge KK, Tsien RY. 2000. The structure of the chromophore within DsRed, a red fluorescent protein from coral. Proc Natl Acad Sci U S A 97:11990-5.
